# Supplementary material for: Parental-perceived home and neighborhood environmental correlates of accelerometer-measured physical activity among school-going children in Uganda
Source: PLOS Glob Public Health. 2021 Dec 8;1(12):e0000089. doi: 10.1371/journal.pgph.0000089 (PMC10021676; doi:10.1371/journal.pgph.0000089)
Supplement: S1 Table — (DOCX) [file pgph.0000089.s001.Docx]

# Description of Parent Perceptions of Home and Neighbourhood Environment attributes

1. **Parents’ Perceptions of the Home Built-Environment**
2. **Social Support**

Description of parents perceived social support.

| Social Support | N=400* | | N=256** | |
| --- | --- | --- | --- | --- |
|  | Low  n (%) | High n (%) | Low  n (%) | High  n (%) |
| Watched their child participate in physical activity | 216 (54.0) | 184 (46.0) | 143 (55.9) | 113 (44.1) |
| Encouraged their child to do sports or physical activity | 167 (41.8) | 233 (58.3) | 110 (43.0) | 146 (57.0) |
| Provided transport to a place where their child can do physical activity or sports | 284(71.0) | 116 (29.0) | 176 (68.8) | 80 (31.3) |
| Did physical activity or played sport with their child | 304(76.0) | 96 (24.0) | 190 (74.2) | 66 (25.8) |

1. **Rules for Physical Activity**

Counts and percentages of parents’ rules for physical activity.

| Rules | N=400* | N=256** |
| --- | --- | --- |
|  | Yes, n (%) | Yes, n (%) |
| Stay close or within sight of the house/parent | 384(96.0) | 245 (95.7) |
| Do not go into the street | 361 (90.3) | 228 (89.1) |
| Come in before dark | 369 (92.3) | 234 (91.4) |
| Do not go to places alone | 366 (91.5) | 236 (92.2) |
| Stay in the neighbourhood | 250 (62.5) | 162 (63.3) |
| Do not ride the bike on the street | 313 (78.3) | 207 (80.9) |
| Do homework before going out | 369 (92.3) | 240 (93.8) |
| Watch out for cars | 347 (86.8) | 226 (88.3) |
| Check in frequently | 332 (83.0) | 218 (85.2) |
| Stay on paths, trails or sidewalks | 328 (82.0) | 215 (84.0) |
| Do not cross busy streets | 358 (89.5) | 236 (92.2) |
| No TV/DVD/computer before homework | 350 (87.5) | 228 (89.1) |
| Less than 2 hours of TV/DVD/computer per day | 251 (62.7) | 158 (61.7) |
| Do not fight with other children | 385 (96.3) | 248 (96.8) |
| Respect others (particularly adults) | 374 (93.5) | 243 (94.9) |

*Note.* TV: Television, DVD: Digital Video Disk, n=Total, *full sample, **sample with accelerometer data

1. **Children’s Media and Electronics Equipment at Home**

Counts and percentages of children’s electronic and media equipment at home.

| Bedroom electronic and media equipment | N=400* | N=256** |
| --- | --- | --- |
|  | Yes n (%) | Yes n (%) |
| TV | 48 (12.0) | 30 (11.7) |
| VCR or DVD player | 33 (8.3) | 23 (9.0) |
| Computer | 53 (13.3) | 31 (12.1) |
| Video game system (non-handheld – play station, x-box, etc. | 61 (15.3) | 40 (15.6) |
| Personal Electronic and media equipment |  |  |
| Cell phone / 2-way radio | 68 (17) | 42 (16.4) |
| Handheld video game players (game boy, Sony PSP, etc. | 73 (18.3) | 55 (21.5) |
| A music player (radio, CD or tape player, stereo, MP3 or I Pod) | 66 (16.5) | 47 (18.4) |

Note. TV: Television, DVD: Digital Video Disk, VCR: Video Cassette recorder., CD: Compact Disk, n=Total, *full sample, **sample with accelerometer data

1. **Play Equipment at Home**

Counts and percentages of play equipment at home.

| Play equipment | N=400* | N=256** |
| --- | --- | --- |
|  | Yes n (%) | Yes n (%) |
| 1. Bike | 190 (47.5) | 136(53.1) |
| 1. Basketball hoop | 18 (4.5) | 14 (5.5) |
| 1. Jump rope | 293 (73.3) | 187(73.0) |
| 1. Active video games (e.g., with dance pad, Wii) | 112 (28.0) | 78 (30.5) |
| 1. Sports equipment (e.g., balls, racquets, bats, sticks) | 295 (73.8) | 192(75.0) |
| 1. Roller skates, skateboard, scooter | 66 (16.5) | 52 (20.3) |
| 1. Fixed play equipment (e.g., swing set, playhouse, gym) | 62 (15.5) | 43 (16.8) |
| 1. Home aerobic equipment (e.g., treadmill, cycle, cross trainer, stepper, rower, workout video or audiotapes) | 60 (15.0) | 40 (15.6) |
| 1. Weightlifting equipment toning devices (e.g., free weights, pull, up bars, exercise balls, ankle weights | 51 (12.8) | 39 (15.2) |
| 1. Yoga/exercise mats | 79 (19.8) | 51 (19.9) |
| 1. Exercise, play or recreation room | 81 (20.3) | 54 (21.1) |
| 1. Trampoline | 19 (4.8) | 14 (5.5) |
| 1. Stairs | 132 (33.0) | 95 (37.1) |

Note. TV: Television, DVD: Digital Video Disk, VCR: Video Cassette recorder, CD: Compact Disk, n=Total, *full sample, **sample with accelerometer data

1. **Parents’ Perceptions of the Neighbourhood Built Environment**
2. **Residential Density**

Counts and percentages of parents’ perceptions of residential density.

| Characteristics | N=400* | N=256** |
| --- | --- | --- |
|  | n (%) | n (%) |
| 1. Very few residential buildings/dwellings within 2 to 5 minutes’ walk of my house | 88 (22) | 49 (19.1) |
| 1. Detached or semi-detached-single-family houses with space/garden | 107 (26.8) | 81 (31.6) |
| 1. Attached (row) housing, apartment blocks/flats or multi-family housing with 2 to 5 storeys | 69 (17.3) | 51 (19.9) |
| 1. Multiple apartment blocks/flats of 6 stories or more with large spaces between buildings. | 30 (7.5) | 23 (9.0) |
| 1. Multiple apartments blocks/flats of 6 stories or more with little space between buildings | 7 (1.8) | 3 (1.2) |
| 1. Very densely packed small houses (1-storey homes, including informal settlements and slums) | 99 (24.8) | 19 (19.1) |

N=grand total, n= subtotal, *full sample, **sample with accelerometer data

1. **Land Use Mix-Diversity (Destinations)**

Counts and percentages of parents’ perceptions of land use mix-diversity (destinations).

| Characteristic | N=400* | | N=256** | |
| --- | --- | --- | --- | --- |
|  | 1 to 20 minutes n (%) | >21 minutes n (%) | 1 to 20 minutes  n (%) | >21 minutes  n (%) |
| 1. Kiosk/corner store | 340 (85.0) | 60 (15.0) | 219 (85.5) | 37 (14.5) |
| 1. Supermarket | 250 (62.5) | 150 (37.5) | 161 (62.9) | 95 (37.1) |
| 1. Fruit/vegetable market (food market) | 243 (60.8) | 157 (39.3) | 149 (58.2) | 107 (41.8) |
| 1. Fast food restaurant | 187 (46.8) | 213 (53.3) | 118 (46.1) | 138 (53.9) |
| 1. Non-fast-food restaurant | 184 (46.0) | 216 (54.0) | 121 (47.3) | 135 (52.7) |
| 1. Pub or bar | 180 (45.0) | 220 (55.0) | 121 (47.3) | 135 (52.7) |
| 1. Cinema or theatre | 87 (21.8) | 313 (78.3) | 58 (22.7) | 198 (77.3) |
| 1. Place of worship/faith centre (church, mosque) | 194 (48.5) | 206 (51.5) | 126 (49.2) | 130 (50.8) |
| 1. Computer/cell phone kiosks/places for internet | 202 (50.6) | 197 (49.4) | 130 (51.0) | 125 (49.0) |
| 1. Library | 88 (22.0) | 312 (78.0) | 58 (22.7) | 198 (77.3) |
| 1. Any school | 224 (56.0) | 176 (44.0) | 144 (56.3) | 112 (43.8) |
| 1. Your workplace or school (if a student) | 97 (24.3) | 303 (75.8) | 57 (22.3) | 199 (77.7) |
| 1. Book store/book shop | 98 (24.5) | 302 (75.5) | 64 (25.0) | 192 (75.0) |
| 1. Health care clinic/hospital | 201 (50.2) | 199 (49.8) | 125 (48.8) | 131 (51.2) |
| 1. Pharmacy/chemist | 210 (52.5) | 190 (47.5) | 139 (54.3) | 117 (45.7) |
| 1. Saloon/barbershop (hairdresser) | 275 (68.8) | 125 (31.3) | 181 (70.7) | 75 (29.3) |
| 1. Clothing store (tailoring/ fashion/designer shop) | 232 (58.0) | 168 (42.0) | 157 (61.3) | 99 (38.7) |
| 1. Electronic shop | 184 (46.0) | 216 (54.0) | 115 (44.9) | 141 (55.1) |
| 1. Public bus or train stop | 182 (45.5) | 218 (54.5) | 119 (46.5) | 137 (53.5) |
| 1. Taxi or motorbike stop | 280 (70.0) | 120 (30.0) | 182 (71.1) | 74 (28.9) |
| 1. Tap/well water, pond, river or stream | 378 (94.5) | 22 (5.5) | 242 (94.5) | 14 (5.5) |

*Note*. N=grand total, n= subtotal, *full sample, **sample with accelerometer data

1. **Land Use Mix-Diversity (Recreation)**

Descriptive statistics of parents’ perceptions of land use mix-diversity (recreation).

| Characteristics | N=400* | | N=256** | |
| --- | --- | --- | --- | --- |
|  | 1 to 20 minutes  n (%) | >21  minutes  n (%) | 1 to 20 minutes  n (%) | >21 minutes  n (%) |
| 1. Sports field or court for basketball, soccer, tennis | 140 (35.0) | 260 (65) | 91 (35.5) | 165 (64.5) |
| 1. Other outdoor recreation facilities (park, open space, information play/recreation | 129 (32.3) | 271 (67.8) | 91 (35.5) | 165 (64.5) |
| 1. Other indoor recreation facilities (recreation centre, gymnasium, health and fitness centre | 119 (29.8) | 281 (70.3) | 91 (35.5) | 165 (64.5) |
| 1. Dance and martial arts classes (karate) | 74 (18.5) | 326 (81.5) | 59 (23.0) | 197 (77.0) |

*Note.* N=grand total, n= subtotal, *full sample, **sample with accelerometer data

1. **Land Use Mix-Access**

Description of parents’ perceptions of land use mix-access.

| Characteristics | N=400* | | N=256** | |
| --- | --- | --- | --- | --- |
|  | Agree n (%) | Disagree  n (%) | Agree n (%) | Disagree  n (%) |
| Stores (shops) are within walking distance of my house | 323 (80.8) | 77 (19.3) | 211 (82.4) | 45 (17.6) |
| There are many places to go such as food markets and restaurants within easy walking distance from my house | 280 (70.0) | 120 (30.0) | 182 (71.1) | 74 (28.9) |
| It is easy to walk to a transit/transport stop (bus, taxi, motorbike, tricycle, train) from my house. | 310 (77.5) | 90 (22.5) | 199 (77.7) | 57 (22.3) |
| It is easy to walk to an outdoor recreation play space (park, open space, informal play/recreation area) from my house | 198 (49.5) | 202 (50.5) | 131 (51.2) | 125 (48.8) |
| It is easy to walk to an indoor recreation facility (recreation centre, gymnasium, health and fitness centre) from my house. | 168 (42.0) | 232 (58.0) | 109 (42.6) | 147 (57.4) |
| The place to get essential supplies like water and firewood are within easy walking distance of my house | 280 (70.0) | 120 (30.0) | 179 (69.9) | 77 (30.1) |
| There are gathering places (community centre, king place, village square, church/worship places etc.) within easy distance | 298 (74.5) | (25.5) | 188 (73.4) | 68 (26.6) |

*Note.* N=grand total, n= subtotal, *full sample, **sample with accelerometer data

1. **Street Connectivity**

Descriptive statistics of parents’ perceptions of street connectivity.

| Characteristics | N=400* | | N=256** | |
| --- | --- | --- | --- | --- |
|  | Agree  n (%) | Disagree  n (%) | Agree  n (%) | Disagree n (%) |
| The distance to walk to the (closest) next street in my neighbourhood is usually short (100 meters or less; the length of a football field). | 292 (73.0) | 108 (27.0) | 190 (74.2) | 66 (25.8) |
| There are many (3 or more) alternative roads (official routes) for getting from place to place in my neighbourhood (I do not have to go the same way all the time). | 285 (71.3) | 115 (28.7) | 186 (72.7) | 70 (27.3) |
| There are many (3 or more) unofficial routes (walking/footpaths) connecting places. | 278 (69.5) | 122 (30.5) | 176 (68.8) | 80 (31.3) |
| There are many (3 or more) shortcuts such as footpaths between roads (official routes) in my area. | 256 (64.0) | 144 (36.0) | 155 (60.5) | 101 (39.5) |
| Some roads (official routes) or walking/footpaths (unofficial routes) in my area are blocked by gates or barriers. | 205 (51.4) | 194 (48.6) | 123 (48.2) | 131 (51.8) |

*Note.* N=grand total, n= subtotal, *full sample, **sample with accelerometer data

1. **Walking and Cycling Infrastructure**

Descriptive statistics of parents’ perceptions of walking and cycling infrastructure.

| Characteristics | N=400* | | N=256** | |
| --- | --- | --- | --- | --- |
|  | Agree  n (%) | Disagree  n (%) | Agree  n (%) | Disagree  n (%) |
| There are formally provided sidewalks (pedestrian pavements) on most of the roads (official routes) in my neighbourhood. | 200 (50.0) | 200 (50.0) | 125 (48.8) | 131 (51.2) |
| The sidewalks in my neighbourhood are well maintained (paved, even, and with less cracks). | 146 (36.5) | 254 (63.5) | 94 (36.7) | 162 (63.3) |
| The sidewalks in my neighbourhood are often blocked by merchandise, construction materials, parked cars, and gardens/lawns/barricades. | 223 (55.8) | 177 (44.3) | 141 (55.1) | 115 (44.9) |
| Sidewalks are separated from the road (vehicle traffic) in my neighbourhoods by parked cars or dedicated parking bays/curbs. | 133 (33.3) | 267 (66.8) | 82 (32.0) | 174 (68.0) |
| There is grass/dirt strip that separates the road from the sidewalks in my neighbourhoods. | 155 (38.8) | 244 (61.2) | 93 (36.5) | 162 (63.5) |
| There are signals or crosswalks/zebra crossings to help walkers cross the busy roads in my neighbourhood. | 145 (36.3) | 255 (63.7) | 84 (32.8) | 172 (67.2) |
| There are curb ramps (decline or smooth grades) that go from sidewalks level to road level at road crossings (intersections /junctions) in my neighbourhood that assist the elderly, wheelchair/pram users. | 107 (26.8) | 293 (73.3) | 62 (24.2) | 194 (75.8) |
| There is enough time for people on foot to cross the road at crossing points/junctions with traffic lights, signals or robots. | 164 (41.1) | 236 (58.9) | 97 (38.0) | 158 (62.0) |
| In my neighbourhood/area there are busy roads that are dangerous to cross. | 176 (44.0) | 224 (56.0) | 113 (44.1) | 143 (55.9) |
| There are informal places (walk/footpaths) for people to walk in my neighbourhood. | 281 (70.3) | 119 (29.8) | 174 (68.0) | 82 (32.0) |
| The walk/footpaths in my neighbourhood are generally of good quality (few potholes, ditches, un-evenness, stones, obstructions), so it is not difficult to walk there. | 177 (44.3) | 223 (55.8) | 111(43.4) | 145 (56.6) |
| There are designated or marked places to bicycle, such as separate paths or trails or shared-use paths for cyclists and pedestrians in or near my neighbourhood. | 102(25.5) | 298(74.5) | 56 (21.9) | 200 (78.1) |

*Note*. N=grand total, n= subtotal, *full sample, **sample with accelerometer data

1. **Neighbourhood Surroundings/Aesthetics**

Descriptive statistics of parents’ perceptions of aesthetics

| Characteristics | N=400* | | N=256** | |
| --- | --- | --- | --- | --- |
|  | Agree n (%) | Disagree n (%) | Agree n (%) | Disagree n (%) |
| There are trees along the roads/paths in my neighbourhood. | 219 (54.8) | 181 (45.3) | 143 (55.9) | 113 (44.1) |
| My neighbourhood is clean and free of litter, garbage or stagnant water. | 202 (50.5) | 198 (49.5) | 124 (48.4) | 132 (51.6) |
| My neighbourhood is free of bad smell and odours. | 252 (63.0) | 148 (37.0) | 162 (63.3) | 94 (36.7) |
| There are beautiful natural sights/views in my neighbourhood. | 228 (57.0) | 172 (43.0) | 144 (56.3) | 112 (43.8) |
| There are attractive buildings/houses in my neighbourhood. | 312 (78.0) | 88 (22.0) | 196 (76.6) | 60 (23.4) |
| My neighbourhood is generally free of unpleasant noises like highways, factories, trains, bars, music/record studios, night clubs/discotheques etc. | 219 (54.8) | 181 (45.3) | 142 (55.5) | 114 (44.5) |
| My neighbourhood is generally free of noticeable pollution and dust, e.g., from traffic or factories. | 235 (58.8) | 165 (41.3) | 145 (56.6) | 111 (43.4) |
| There are many pleasant natural sounds in my neighbourhood e.g., from birds | 241(60.4) | 158 (39.6) | 159 (62.1) | 97 (37.9) |

*Note.* N=grand total, n= subtotal, *full sample, **sample with accelerometer data

1. **Crime Safety**

Counts and percentages of parents’ perceptions of crime safety.

| Characteristics | N=400* | | N=256** | |
| --- | --- | --- | --- | --- |
|  | Agree  n (%) | Disagree  n (%) | Agree  n (%) | Disagree  n (%) |
| There is a lot of crime rate in my neighbourhood. | 244(61.0) | 156 (39.0) | 160 (62.5) | 96 (37.5) |
| There is too much crime in my neighbourhood to go outside for walks or play during the day. | 165(41.3) | 235 (58.8) | 105 (41.1) | 57 (58.9) |
| There is too much crime in my neighbourhood to go outside for walks or play at night. | 303(75.8) | 97 (24.3) | 187 (73.0) | 69 (27.0) |
| There are groups of people or gangs (rascals, hooligans, and thugs) in my neighbourhood who make me feel threatened when I go out. | 239(59.8) | 161 (40.3) | 155 (60.5) | 101 (39.5) |

*Note.* N=grand total, n= subtotal, *full sample, **sample with accelerometer data

1. **Traffic Safety**

Descriptive statistics of parents’ perceptions of traffic safety

|  | N=400* | | N=256** | |
| --- | --- | --- | --- | --- |
| Characteristics | Agree  n (%) | Disagree  n (%) | Agree  n (%) | Disagree  n (%) |
| There is so much traffic along nearby roads that it is difficult or unpleasant to either walk or play in my neighbourhood. | 212 (53.0) | 188 (47.0) | 140 (54.7) | 116 (45.3) |
| The speed of traffic on most nearby roads in my neighbourhood is usually slow. | 197 (49.3) | 203 (50.7) | 129 (50.4) | 127 (49.6) |
| Most drivers exceed the speed limits (drive extremely fast) in my neighbourhood. | 187 (46.5) | 214 (53.5) | 125 (48.8) | 131 (51.2) |
| Walking or playing is dangerous in my neighbourhood because of careless or aggressive driving. | 186 (46.8) | 213 (53.3) | 128 (50.0) | 128 (50.0) |
| It could be dangerous to ride on a bicycle in or near my neighbourhood because of the speed of traffic. | 195 (48.8) | 204 (51.2) | 137 (53.5) | 119 (46.5) |
| I am worried about playing or walking in my neighbourhood and local streets because I am afraid of being injured by a car. | 181 (45.4) | 218 (54.6) | 119 (46.7) | 136 (53.3) |

*Note.* N=grand total, n= subtotal, *full sample, **sample with accelerometer data

1. **Personal Safety**

Counts and percentages of parents’ perceptions of personal safety.

| Characteristics | N=400* | | N=256** | |
| --- | --- | --- | --- | --- |
|  | Agree  n (%) | Disagree  n (%) | Agree  n (%) | Disagree  n (%) |
| I see, and I can talk to people when I am walking in my neighbourhood. | 349 (87.3) | 51 (12.8) | 224 (87.5) | 32 (12.5) |
| There are stray dogs or dangerous animals that scare me in my neighbourhood. | 237 (59.3) | 163 (40.8) | 147 (57.4) | 109 (42.6) |
| The roads in my neighbourhood are well lit (adequate functioning streetlights) at night. | 143 (35.8) | 257 (64.3) | 92 (35.9) | 164 (64.1) |

*Note.* N=grand total, n= subtotal, *full sample, **sample with accelerometer data

1. **Stranger Danger**

Counts and percentages of parents’ perceptions of stranger danger.

| Characteristics | N=400* | | N=256** | |
| --- | --- | --- | --- | --- |
|  | Agree  n (%) | Disagree  n (%) | Agree  n (%) | Disagree  n (%) |
| I am worried about letting my child play or staying outside alone or with friends around my house (e.g., yard, driveway, apartment common area) because I am afraid, he/she might be taken or hurt by a stranger. | 140 (35.0) | 260 (65.0) | 168(34.4) | 88 (65.6) |
| I am worried about letting my child play or walk alone or with friends in my neighbourhood and local streets because I am afraid, he/she being taken or hurt by a stranger. | 125 (31.3) | 275 (68.8) | 74 (28.9) | 182 (71.1) |
| I am worried about letting my child to be alone or with friends in a local or nearby park because I am afraid of, he/she being taken or hurt by a stranger. | 117 (29.3) | 282 (70.7) | 68 (26.7) | 187 (73.3) |

*Note*. N=grand total, n= subtotal, *full sample, **sample with accelerometer data
