# Supplementary material for: Parental-perceived home and neighborhood environmental correlates of accelerometer-measured physical activity among school-going children in Uganda
Source: PLOS Glob Public Health. 2021 Dec 8;1(12):e0000089. doi: 10.1371/journal.pgph.0000089 (PMC10021676; doi:10.1371/journal.pgph.0000089)
Supplement: S1 File — (DOCX) [file pgph.0000089.s002.docx]

**PARENT/GUARDIAN QUESTIONNAIRE**

We would like to learn more about you, your child, your home and neighborhood. Please answer all questions as accurate as you can. Remember that there are no right or wrong answers, and that every person is different. We will not share any of your personal information with anyone else, and all of your answers will remain private.

**Instructions**

Please try not to miss any questions. Provide only one answer for each question, using black or blue ink.

**SECTION A: SOCIO-DEMOGRAPHIC INFORMATION**

***Please respond to these questions for the child who is participating in the study***

1. Your child’s date of birth ___________________
2. Child’s sex:

0. 🞎 Male

1. 🞎 Female

***Please respond to these questions about yourself.***

1. Your age: ________ years
2. Your sex:

0. 󠄋**🞎**Male

1. 󠆥 **🞎**󠆥 Female

1. What is your marital status?

1. 󠄋**🞎** Married

2. 󠆥**🞎** Widowed/divorced

3. **🞎** Single

4. 󠄋**🞎** Living with partner

1. What was the highest education level you completed?
2. 🞎 Primary
3. 🞎 O level certificate
4. 🞎 A level certificate
5. 🞎󠄋 Diploma
6. 🞎󠆥 Bachelor’s degree
7. 🞎󠄋 Postgraduate degree
8. How many functioning motorized transport / vehicles (cars, motorcycles/bikes, tricycles, and trucks) are available for use in your household?
9. **🞎** None
10. **🞎** One
11. **🞎** Two
12. **🞎** Three or more
13. How long have you lived at your present address (house)? ________years and _____months.
14. How many children and youth (aged 0-17 years) live in your household most of the time? ______youth.

**Think about your child’s activities over the PAST YEAR as you answer the following questions, unless otherwise specified.**

**SECTION B: QUESTIONS ABOUT YOUR HOME**

***Please put a check mark (√) in the box besides the answer that best applies to you, your child and your home*.**

| ***Parental Support***  **During a typical week, how often have you or another adult in the household:** | **Never** | **1-2 days** | **3-4 days** | **5-6 days** | **Every day** |
| --- | --- | --- | --- | --- | --- |
| 1. Watched your child participate in physical activity or sports | 🞎 | 🞎 | 🞎 | 🞎 | 🞎 |
| 2. Encouraged your child to do sports or physical activity | 🞎 | 🞎 | 🞎 | 🞎 | 🞎 |
| 3. Provided transport to a place where your child can do physical activity or play sports | 🞎 | 🞎 | 🞎 | 🞎 | 🞎 |
| 4. Done a physical activity or played sports with your child | 🞎 | 🞎 | 🞎 | 🞎 | 🞎 |

| **Parent Rules**  **Which of the following rules do you enforce about your child’s activity?** | **Yes** | **No** |
| --- | --- | --- |
| 1. Stay close to or within sight of the house/parent | 🞎 | 🞎 |
| 2. Do not go into the street | 🞎 | 🞎 |
| 3. Return home before dark | 🞎 | 🞎 |
| 4. Do not go places alone | 🞎 | 🞎 |
| 5. Stay in the neighborhood | 🞎 | 🞎 |
| 6. Do not ride bike on the street | 🞎 | 🞎 |
| 7. Do homework before going out | 🞎 | 🞎 |
| 8. Watch out for cars | 🞎 | 🞎 |
| 9. Check in frequently | 🞎 | 🞎 |
| 10. Stay on paths, trails or sidewalk | 🞎 | 🞎 |
| 11. Do not cross busy streets | 🞎 | 🞎 |
| 12. No TV/DVD/computer before homework | 🞎 | 🞎 |
| 13. Less than 2 hours TV/DVD/computer per day | 🞎 | 🞎 |
| 14. Do not fight with other kids | 🞎 | 🞎 |
| 15. Do not disrespect others (particularly adults) | 🞎 | 🞎 |
| **Things in Your Child’s Bedroom**  **Please indicate (√) whether the following are in your child’s bedroom.** | | |
| 1. TV | Yes | No |
| 2. VCR or DVD player | Yes | No |
| 3. Music players (radio, CD or tape player, stereo, MP3 or iPod) | Yes | No |
| 4. Computer | Yes | No |
| 5. Video game system (non-hand held—Play station, x-box, etc.) | Yes | No |
| **Your Child’s Personal Electronics**  **Does your child have the following items for his/her own use?** | | |
| 6. Cell phone or 2-way radio | Yes | No |
| 7. Hand held videogame players (game boy, Sony PSP, etc.) | Yes | No |

| ***Play Equipment***  **Please indicate (√) if you have the following items in your home, yard or apartment complex, and if you have them, how often your child uses each item. Please circle the answer that best applies to your child.** | | | | | |
| --- | --- | --- | --- | --- | --- |
|  | **Not Available (I Don’t Have)** | **Available but never used** | **Once a month or less** | **Once every other week** | **Once a week or more** |
| 1. Bike | **🞎** | **🞎** | **🞎** | **🞎** | **🞎** |
| 1. Basketball hoop | **🞎** | **🞎** | **🞎** | **🞎** | **🞎** |
| 1. Jump rope | **🞎** | **🞎** | **🞎** | **🞎** | **🞎** |
| 1. Active video games (e.g. with dance pad, Wii, etc.) | **🞎** | **🞎** | **🞎** | **🞎** | **🞎** |
| 1. Sports equipment (e.g. balls, racquets, bats, sticks) | **🞎** | **🞎** | **🞎** | **🞎** | **🞎** |
| 1. Roller skates, skateboard, scooter | **🞎** | **🞎** | **🞎** | **🞎** | **🞎** |
| 1. Fixed play equipment (e.g., swing set, play house, gym) | **🞎** | **🞎** | **🞎** | **🞎** | **🞎** |
| 1. Home aerobic equipment (e.g. treadmill, cycle, cross trainer stepper, rower, workout video or audio tapes) | **🞎** | **🞎** | **🞎** | **🞎** | **🞎** |
| 1. Weight lifting equipment, toning devices (e.g., free weights, pull, up bars, exercise balls, ankle weights etc.) | **🞎** | **🞎** | **🞎** | **🞎** | **🞎** |
| 1. Yoga/exercise mats | **🞎** | **🞎** | **🞎** | **🞎** | **🞎** |
| 1. Exercise, play or recreation room | **🞎** | **🞎** | **🞎** | **🞎** | **🞎** |
| 1. Trampoline | **🞎** | **🞎** | **🞎** | **🞎** | **🞎** |
| 1. Stairs | **🞎** | **🞎** | **🞎** | **🞎** | **🞎** |

**SECTION C: QUESTIONS ABOUT YOUR NEIGHBORHOOD**

We would like to find out more information about what you perceive or think and how you feel about your neighborhood. By neighborhood we mean **ALL** the areas that you could walk to in **10 to 20** **minutes** **from your house** (within approximately one kilometer or half a mile of your house). Please check (√) the answer that best applies to you and your neighborhood.


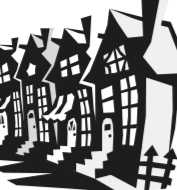
**A. Types of residences in your neighborhood**

***Please put a check mark (√) in the box beside the answer that best applies to you and your neighborhood (please select only one answer).***

1.What is the main type of housing in your immediate neighborhood?

1. **🞎** Very few residential buildings/dwellings within 2 to 5 min walk of my house
2. **🞎** Detached or semi-detached single-family houses with space/garden
3. **🞎** Attached (row) housing, apartment blocks/flats or multi-family housing with 2 to 5 storeys.
4. **🞎**Multiple apartment blocks/flats of 6 stories or more, with large spaces between buildings.
5. **🞎**Multiple apartment blocks/flats of 6 storeys or more, with very little space between buildings.
6. **🞎** Very densely packed small houses (1-storey homes, including informal settlements and slums

**
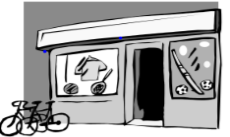
**

**B. Stores, facilities, and other things in your neighborhood**

Please think about one common destination you go to from your house very often, and how many minutes does it take you? Thinking about how long it takes you to walk to this destination can help you with the next questions. ***Approximately how long would it take you to walk from your house to the nearest places or locations listed below. Please put only one check mark (√) for each business or facility.***

|  | **1-5 min** | **6-10 min** | **11-20 min** | **21-30 min** | **31+ min** | **I Don’t**  **know** |
| --- | --- | --- | --- | --- | --- | --- |
| Example: gas / petrol station | 1 | 2 | 3 √ | 4 | 5 | 8 |
| 1. Kiosk/corner store/small grocery | 1 | 2 | 3 | 4 | 5 | 8 |
| 1. Supermarket | 1 | 2 | 3 | 4 | 5 | 8 |
| 1. Fruit / vegetable market (food Market) | 1 | 2 | 3 | 4 | 5 | 8 |
| 1. Fast food restaurant | 1 | 2 | 3 | 4 | 5 | 8 |
| 1. Non-fast food restaurant | 1 | 2 | 3 | 4 | 5 | 8 |
| 1. Pub or bar | 1 | 2 | 3 | 4 | 5 | 8 |
| 1. Cinema or theatre | 1 | 2 | 3 | 4 | 5 | 8 |
| 1. Place of worship / faith center (church, Mosque, Shrine) | 1 | 2 | 3 | 4 | 5 | 8 |
| 1. Computer / cell phone kiosks. places for internet or phone calls | 1 | 2 | 3 | 4 | 5 | 8 |
| 1. Library | 1 | 2 | 3 | 4 | 5 | 8 |
| 1. Any school | 1 | 2 | 3 | 4 | 5 | 8 |
| 1. Your work place or your school (if a student) | 1 | 2 | 3 | 4 | 5 | 8 |
| 1. Book store / book shop | 1 | 2 | 3 | 4 | 5 | 8 |
| 1. Health care clinic / hospital | 1 | 2 | 3 | 4 | 5 | 8 |
| 1. Pharmacy / chemist | 1 | 2 | 3 | 4 | 5 | 8 |
| 1. Salon / barber shop (hair dresser) | 1 | 2 | 3 | 4 | 5 | 8 |
| 1. Clothing store (Tailoring / fashion / designer shop) | 1 | 2 | 3 | 4 | 5 | 8 |
| 1. Electronic shop | 1 | 2 | 3 | 4 | 5 | 8 |
| 1. Public bus or train stop | 1 | 2 | 3 | 4 | 5 | 8 |
| 1. Taxi or motorbike stop | 1 | 2 | 3 | 4 | 5 | 8 |
| 1. Sports field or court for basketball, soccer, tennis etc. | 1 | 2 | 3 | 4 | 5 | 8 |
| 1. Other outdoor recreation facilities (park, open space, informal play / recreation area) | 1 | 2 | 3 | 4 | 5 | 8 |
| 1. Other indoor recreation facilities (recreation center, gymnasium, health and fitness center) | 1 | 2 | 3 | 4 | 5 | 8 |
| 1. Dance and martial arts classes (karate) | 1 | 2 | 3 | 4 | 5 | 8 |
| 1. Tap/well water, pond, river or stream for fresh water (if plumbing is in house choose “1-5” minutes) | 1 | 2 | 3 | 4 | 5 | 8 |


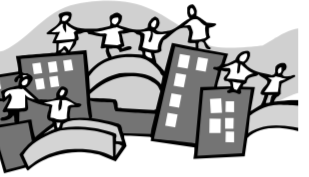
 **C. Access to services and places**

*Please circle the answer that best applies to the neighborhood where you and your child live. Both local and within walking distance mean within a 10-15-minute walk from your house.*

1. Stores (shops) are within easy walking distance of my house.

1 2 3 4

strongly somewhat somewhat strongly

disagree disagree agree agree

1. There are many places to go such as food markets and restaurants within easy walking distance of my house.

1 2 3 4

strongly somewhat somewhat strongly

disagree disagree agree agree

1. It is easy to walk to a transit / transport stop (bus, taxi, motorbike, tricycle, train) from my house.

1 2 3 4

strongly somewhat somewhat strongly

disagree disagree agree agree

1. It is easy to walk to an outdoor recreation play space (park, open space, informal play / recreation area) from my house.

1 2 3 4

strongly somewhat somewhat strongly

disagree disagree agree agree

1. It is easy to walk to an indoor recreation facility (recreation center, gymnasium, health and fitness center) from my house.

1 2 3 4

strongly somewhat somewhat strongly

disagree disagree agree agree

1. Places to get essential supplies like water and firewood are within easy walking distance of my house.

1 2 3 4

strongly somewhat somewhat strongly

disagree disagree agree agree

1. There are gathering places (community center, king place, village square, church / worship places etc.) within easy distance of my house.

1 2 3 4

strongly somewhat somewhat strongly

disagree disagree agree agree


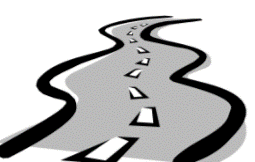
**D. Roads and walking paths**

*Please circle the answer that best applies to you and your neighbourhood.*

1. The distance to walk to the (closest) next street in my neighborhood is usually short (100 meters or less; the length of a football field or less).

1 2 3 4

strongly somewhat somewhat strongly

disagree disagree agree agree

1. There are many (3 or more) alternative roads (official routes) for getting from place to place in my neighbourhood (I don’t have to go the same way all the time).

1 2 3 4

strongly somewhat somewhat strongly

disagree disagree agree agree

1. There are many (3 or more) unofficial routes (walking / foot paths) connecting places in my area.

1 2 3 4

strongly somewhat somewhat strongly

disagree disagree agree agree

1. There are many (3 or more) shortcuts such as foot paths between roads (official routes) in my area.

1 2 3 4

strongly somewhat somewhat strongly

disagree disagree agree agree

1. Some roads (official routs) or walking / foot paths (unofficial routes) in my area are blocked by gates or barriers.

1 2 3 4

strongly somewhat somewhat strongly

disagree disagree agree agree


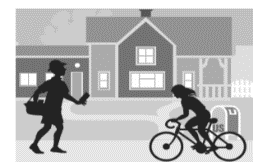
**E. Places for walking, cycling and playing**

*Please circle the answer that best applies to you and your neighbourhood.*

1. There are formally provided sidewalks (pedestrian pavements) on most of the roads (official routes) in my neighbourhood.

1 2 3 4

strongly somewhat somewhat strongly

disagree disagree agree agree

1. The sidewalks in my neighborhood are well maintained (paved, even, and not a lot of cracks).

1 2 3 4

strongly somewhat somewhat strongly

disagree disagree agree agree

1. The sidewalks in my neighborhood are often blocked by merchandise, construction materials, parked cars, gardens / lawns / barricades.

1 2 3 4

strongly somewhat somewhat strongly

disagree disagree agree agree

1. Sidewalks are separated from the road (vehicle traffic) in my neighbourhood by parked cars or dedicated parking bays / curbs.

1 2 3 4

strongly somewhat somewhat strongly

disagree disagree agree agree

1. There is grass / dirt strip that separates the road from the sidewalks in my neighbourhood.

1 2 3 4

strongly somewhat somewhat strongly

disagree disagree agree agree

1. There are signals or crosswalks / zebra crossings to help walkers cross the busy roads in my neighborhood.

1 2 3 4

strongly somewhat somewhat strongly

disagree disagree agree agree

1. There are curb ramps (decline or smooth grades) that go from sidewalks level to road level at road crossings (Intersections / junctions) in my neighbourhood that assist the elderly or wheel chair / pram users.

1 2 3 4

strongly somewhat somewhat strongly

disagree disagree agree agree

1. There is enough time for people on foot to cross the road at crossing points / junctions with traffic lights, signals or robots.

1 2 3 4

strongly somewhat somewhat strongly

disagree disagree agree agree

1. There are informal places (Walk / footpaths) for people to walk in my neighbourhood.

1 2 3 4

strongly somewhat somewhat strongly

disagree disagree agree agree

1. The walk / foot paths in my neighborhood are generally of good quality (few potholes, ditches, un-evenness, stones, obstructions), so it is not difficult to walk there.

1 2 3 4

strongly somewhat somewhat strongly

disagree disagree agree agree

1. In my neighborhood / area there are busy roads that are dangerous to cross.

1 2 3 4

strongly somewhat somewhat strongly

disagree disagree agree agree

1. There are designated or marked places to bicycle, such as separate paths or trails or shared use paths for cycle and pedestrians in or near my neighbourhood.

1 2 3 4

strongly somewhat somewhat strongly

disagree disagree agree agree


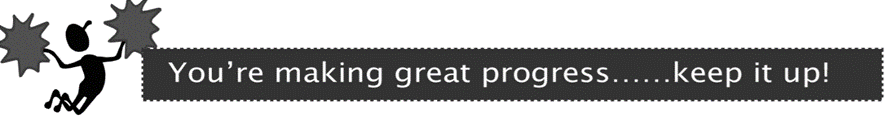


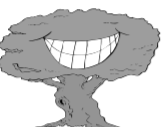


**F. Neighborhood surroundings**

*Please circle the answer that best applies to you and your neighborhood*

1. There are trees along the roads / paths in my neighborhood.

1 2 3 4

strongly somewhat somewhat strongly

disagree disagree agree agree

1. My neighborhood is clean and free of litter, garbage or stagnant water.

1 2 3 4

strongly somewhat somewhat strongly

disagree disagree agree agree

1. My neighbourhood if free from bad smell and adors.

1 2 3 4

strongly somewhat somewhat strongly

disagree disagree agree agree

1. There are beautiful natural sights / views in my neighbourhood.

1 2 3 4

strongly somewhat somewhat strongly

disagree disagree agree agree

1. There are attractive buildings / houses in my neighbourhood.

1 2 3 4

strongly somewhat somewhat strongly

disagree disagree agree agree

1. My neighborhood is generally free of unpleasant noises like highways, factories, trains, bars, music / record studios, night clubs / discotheques etc.

1 2 3 4

strongly somewhat somewhat strongly

disagree disagree agree agree

1. My neighbourhood is generally free of noticeable pollution and dust, such as from traffic or factories.

1 2 3 4

strongly somewhat somewhat strongly

disagree disagree agree agree

1. There are many pleasant natural sounds in my neighbourhood such as from birds.

1 2 3 4

strongly somewhat somewhat strongly

disagree disagree agree agree


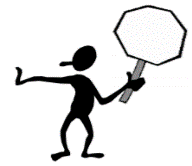


**G. Safety from traffic.**

*Please circle the answer that best applies to you and your neighborhood*

1. There is so much traffic along nearby roads that it is difficult or unpleasant to walk nor play in my neighbourhood.

1 2 3 4

strongly somewhat somewhat strongly

disagree disagree agree agree

1. The speed of traffic on most nearby roads in my neighbourhood is usually slow.

1 2 3 4

strongly somewhat somewhat strongly

disagree disagree agree agree

1. Most drivers exceed the speed limits (drive very fast) in my neighborhood.

1 2 3 4

strongly somewhat somewhat strongly

disagree disagree agree agree

1. Walking or playing is dangerous in my neighborhood because of careless or aggressive driving.

1 2 3 4

strongly somewhat somewhat strongly

disagree disagree agree agree

1. It could be dangerous to ride on a bicycle in or near my neighbourhood because of speed of traffic.

1 2 3 4

strongly somewhat somewhat strongly

disagree disagree agree agree

1. I am worried about playing or walking in my neighbourhood and local streets because I am afraid of being injured by a car.

1 2 3 4

strongly somewhat somewhat strongly

disagree disagree agree agree

**H. Safety from crime**

*Please circle the answer that best applies to you and your neighborhood*

1. There is a lot of crime rate in my neighborhood.

1 2 3 4

strongly somewhat somewhat strongly

disagree disagree agree agree

1. There is too much crime in my neighborhood to go outside for walks or play during the day.

1 2 3 4

strongly somewhat somewhat strongly

disagree disagree agree agree

1. There is too much crime in my neighbourhood to go outside for walks or play at night.

1 2 3 4

strongly somewhat somewhat strongly

disagree disagree agree agree

1. There are groups of people or gangs (rascals, hooligans, and thugs) in my neighbourhood who make me feel threatened when I go out.

1 2 3 4

strongly somewhat somewhat strongly

disagree disagree agree agree

**I. Personal Safety**

*Please circle the answer that best applies to you and your neighborhood.*

1. I see and I can talk to people when I am walking in my neighbourhood.

1 2 3 4

strongly somewhat somewhat strongly

disagree disagree agree agree

1. There are stray dogs or dangerous animals that scare me in my neighbourhood.

1 2 3 4

strongly somewhat somewhat strongly

disagree disagree agree agree

1. The roads in my neighbourhood are well lit (adequate functioning street lights) at night.

1 2 3 4

strongly somewhat somewhat strongly

disagree disagree agree agree

**J. Stranger Danger**

**If you are a parent with a child 17 years old or below, please answer the questions below. When responding to the questions, please think mainly about the child who brought the survey.**

1. I am worried about letting my child *play or being outside alone or with friends* around my house (e.g. yard, driveway, apartment common area), because I am afraid of them being taken or hurt by a stranger.

1 2 3 4

strongly somewhat somewhat strongly

disagree disagree agree agree

1. I am worried about letting my child play or walk *alone or with friends* in my neighborhood and local streets because I am afraid of them being taken or hurt by a stranger.

1 2 3 4

strongly somewhat somewhat strongly

disagree disagree agree agree

1. I am worried about letting my child be alone or *with friends* in a local or nearby park because I am afraid of them being taken or hurt by a stranger.

1 2 3 4

strongly somewhat somewhat strongly

disagree disagree agree agree

***THANK YOU AND WE APPRECIATE YOUR HELP IN COMPLETING THE LONG QUESTIONNAIRE***
