# Supplementary material for: Parental-perceived home and neighborhood environmental correlates of accelerometer-measured physical activity among school-going children in Uganda
Source: PLOS Glob Public Health. 2021 Dec 8;1(12):e0000089. doi: 10.1371/journal.pgph.0000089 (PMC10021676; doi:10.1371/journal.pgph.0000089)
Supplement: S2 File — (PDF) [file pgph.0000089.s003.pdf]

S/N	SCH TYPE	CHD SEX	CHD AGE	TIME AT RESIDENCE	ACCEL. WEAR STATUS	MVPA	WEAR DAYS	WEAR TIME
1	0	0	11	40	1	51.30	6	18.02
2	0	1	12	100	1	31.50	6	19.64
3	0	1	10	117	1	24.60	7	20.01
4	0	1	11	64	2			0.00
5	0	1	11	180	1	45.80	4	14.35
6	0	0	11	69	3			
7	0	0	11	124	1	52.10	7	22.74
8	0	0	12	30	1	26.40	7	17.97
9	0	0	12	180	1	38.60	7	22.23
10	0	1	11	124	1	25.20	7	18.06
11	0	1	11	168	1	34.90	6	21.79
12	0	0	12	160	2			
13	0	0	11	160	1	52.90	5	14.83
14	0	1	10	172	1	35.50	5	14.06
15	0	0	10	150	1	29.50	8	19.09
16	0	1	11	172	1	17.30	5	16.96
17	0	0	12	60	1	25.50	8	21.78
18	0	0	10	96	4			
19	0	0	11	99	1	40.40	8	16.77
20	0	1	12	132	1	60.20	5	15.59
21	0	0	12	86	3			
22	0	0	11	37	1	57.30	8	20.46
23	0	0	11	60	1	48.10	5	13.22
24	0	1	12	32	1	24.80	5	17.94
25	0	1	12	40	3			
26	0	0	12	84	2			
27	0	0	11	136	2			
28	0	1	11	47	1	59.20	6	19.98
29	0	0	11	120	3			
30	0	0	11	240	1	40.20	8	22.56
31	0	1	12	62	5			
32	0	0	11	52	1	35.60	8	22.66
33	0	1	11	144	4			
34	0	0	11	129	1	52.50	7	14.12
35	0	0	12	75	1	23.70	8	21.68
36	0	1	11	56	1	46.00	5	12.23
37	0	0	11	125	1	31.40	7	18.69
38	0	0	11	5	1	55.90	6	14.80
39	0	0	11	148	1	34.60	7	24.00
40	0	1	10	16	1	69.30	6	13.84
41	0	0	11	132	1	42.80	6	14.15
42	0	1	12	72	0			
43	0	1	11	96	1	63.50	6	83.96
44	0	0	11	30	3			
45	0	1	10	66	0			
46	0	1	10	126	0			

47	0	1	11	154	1	14.10	7	15.67
48	0	0	11	72	1	68.00	7	15.10
49	0	0	12	64	3			
50	0	1	11	123	1	36.60	6	15.34
51	0	0	12	91	1	33.40	7	14.57
52	0	0	12	120	1	71.20	6	13.72
53	0	1	11	207	1	23.40	6	15.00
54	0	0	12	72	1	54.60	7	16.03
55	0	1	12	21	1	29.60	8	16.72
56	0	1	12	60	1	43.50	5	13.61
57	0	0	12	141	1	29.60	4	13.23
58	0	1	12	40	1	35.40	6	16.10
59	0	0	12	204	1	72.10	7	14.66
60	0	0	12	144	1	36.70	7	14.56
61	0	1	10	96	1	34.60	5	13.75
62	0	1	11	52	1	29.10	6	14.08
63	0	0	10	120	0			
64	0	1	10	12	2			
65	0	0	12	42	1	72.20	6	14.48
66	0	1	12	76	1	31.10	7	14.71
67	0	1	11	14	1	50.50	6	14.85
68	0	1	12	185	0			
69	0	0	11	51	1	58.70	7	13.75
70	0	1	10	41	1	33.30	7	15.95
71	0	1	12	96	0			
72	0	0	12	104				
73	0	1	10	4	1	31.60	7	14.54
74	0	1	10	68	0			
75	0	0	12	110	1	39.70	7	16.21
76	0	0	12	7	1	26.90	6	14.27
77	0	1	12	53	0			
78	0	0	11	24	1	26.00	7	14.02
79	0	0	10	108	0			
80	0	1	10	62	0			
81	0	1	11	174	2			
82	0	1	12	123	1	27.20	5	12.14
83	0	1	11	184	0			
84	0	0	11	120				
85	0	0	11	6	1	67.10	7	15.56
86	0	1	11	71	1	55.90	6	15.23
87	0	1	12	99	1	18.40	7	16.33
88	0	1	12	129	2			
89	0	1	11	60	1	44.10	6	14.49
90	0	1	12	148	1	31.30	7	16.13
91	0	1	10	184	1	58.20	5	14.86
92	0	0	12	68	4			
93	0	0	10	98	1	45.20	7	14.69

94	0	0	12	148	1	22.70	7	13.23
95	0	1	12	12	1	35.10	8	16.54
96	0	1	12	124	1	38.30	7	14.05
97	0	0	10	168	1	36.90	7	15.18
98	0	1	10	12	2			
99	0	1	11	40	1	27.30	8	15.09
100	0	0	11	65	1	43.30	7	14.06
101	0	1	10	60	1	29.70	5	13.94
102	0	1	12	16				
103	0	0	12	149	1	32.20	5	13.33
104	0	1	11	145	1	28.50	8	14.86
105	0	1	11	143	1	43.50	5	16.26
106	0	0	11	156	1	35.90	7	15.16
107	0	1	11	82	0			
108	0	0	10	251	0			
109	0	0	11	125	0			
110	0	0	11	24	1	36.40	6	14.26
111	0	0	11	60	1	51.50	7	14.34
112	0	1	11	126	1	38.20	4	13.49
113	0	1	11	36	1	41.30	6	14.91
114	0	1	11	12	1	31.20	6	13.46
115	0	1	11	89	1	54.50	5	13.49
116	0	1	11	62	1	32.60	5	17.01
117	0	0	11	94	3			
118	0	1	10	124	1	44.80	7	16.17
119	0	1	12	42	1	25.80	6	14.26
120	0	0	11	120	1	46.50	7	14.44
121	0	0	10	47	0			
122	0	0	12	144	0			
123	0	1	11	300	1	50.00	7	16.02
124	0	0	11	144	1	66.90	7	16.58
125	0	0	11	218	1	64.00	7	14.30
126	0	0	11	166	1	46.10	5	14.13
127	0	0	12	124	1	41.50	7	14.43
128	0	1	11	36				
129	0	0	11	53	1	79.80	5	15.34
130	0	0	10	64	0			0.00
131	0	0	11	12	1	56.50	7	15.19
132	0	0	11	120	0			0.00
133	0	1	12	26	1	71.30	6	13.74
134	0	0	11	24	0			
135	0	1	11	99	1	23.50	7	17.88
136	0	1	12	16	1	52.30	6	15.45
137	0	0	11	156	1	43.20	7	16.83
138	0	1	10	60	0			
139	0	1	10	144	0			
140	0	0	10	120	1	29.60	6	15.43

141	0	1	11	24	1	46.80	5	16.95
142	0	0	10	124	0			0.00
143	0	1	10	53	1	51.70	6	15.48
144	0	0	10	156	1	45.40	8	19.02
145	0	1	10	72	2			
146	0	1	10	19	0			
147	0	1	11	120	1	49.60	6	13.25
148	0	0	12	36	3			
149	0	1	10	18	1	38.70	6	13.27
150	0	1	10	152	0			
151	0	0	12	61	1	47.40	5	15.24
152	0	1	12	62	1	34.80	6	15.06
153	0	0	10	61	1	110.70	5	16.51
154	0	1	11	161	1	44.20	5	13.28
155	0	1	12	146	1	64.00	6	14.65
156	0	0	12	24	1	20.90	9	15.61
157	0	0	10	54	1	48.80	6	15.03
158	0	0	11	30	1	45.80	5	16.63
159	0	0	10	24	3			
160	0	1	11	12	1	47.40	5	15.24
161	0	1	12	87	1	34.80	6	15.06
162	0	1	12	48	1	110.70	5	16.51
163	0	1	10	85	1	44.20	5	13.28
164	0	0	12	112	1	64.00	6	14.65
165	0	0	12	125	1	20.90	9	15.61
166	0	1	11	38	1	48.80	6	15.03
167	0	0	12	172	1	45.80	5	16.63
168	0	1	11	69	1	37.50	5	14.67
169	0	0	11	134	1	100.70	5	14.08
170	0	1	11	74	2			
171	0	1	10	100	1	32.70	6	17.72
172	0	1	12	50	1	40.60	5	14.30
173	0	0	12	96	1	31.00	6	14.22
174	0	0	11	27	1	69.70	5	13.54
175	0	1	11	96	1	65.80	6	14.93
176	0	0	11	240	1	21.00	5	14.58
177	0	1	11	39	1	45.00	7	16.80
178	0	1	10	98	1	62.50	6	14.46
179	0	0	10	24	3			
180	0	0	11	240	1	33.00	7	14.02
181	0	0	11	126	1	83.10	6	14.68
182	0	0	11	84	0			
183	0	1	10	101	1	60.00	6	15.77
184	0	1	10	48	1	50.90	7	14.87
185	0	0	10	48	1	64.70	6	14.80
186	0	0	10	18	3			
187	0	1	11	39	1	47.30	7	16.07

188	0	0	10	48	1	24.00	7	16.14
189	0	0	11	130	1	37.30	6	15.46
190	0	1	11	63	1	49.40	6	14.22
191	0	0	10	50	1	22.70	5	18.46
192	0	1	11	62	1	75.30	5	14.93
193	0	0	11	39	1	51.50	7	16.72
194	0	0	12	51	1	44.80	6	15.60
195	0	1	10	63	1	54.60	7	15.24
196	0	1	10	20	1	60.40	7	14.85
197	0	0	11	14	1	43.80	7	15.16
198	0	0	11	27	1	50.20	6	15.46
199	0	1	10	108	1	43.00	6	14.99
200	0	0	10	48	1	78.40	6	15.38
201	0	1	11	48	1	45.80	4	14.35
202	0	0	10	60	1	50.00	6	15.47
203	0	0	10	108	1	104.80	6	14.74
204	0	0	11	48	1	49.30	6	15.50
205	1	0	12	78	1	83.10	7	15.11
206	1	1	11	34	1	63.90	7	14.85
207	1	1	12	48	1	84.50	6	14.18
208	1	1	12	84	1	47.30	6	15.23
209	1	1	10	36	1	62.90	7	14.96
210	1	1	10	124	1	61.80	7	15.66
211	1	1	11	108	1	33.80	7	15.45
212	1	1	11	84	1	61.10	6	14.86
213	1	0	12	85	1	59.30	7	16.31
214	1	1	11	168	1	40.80	7	14.85
215	1	1	10	36				
216	1	1	10	89	1	77.70	7	16.25
217	1	0	12	39	1	104.20	7	16.11
218	1	1	12	120	3			
219	1	1	11	77	1	55.90	7	13.39
220	1	0	12	87	1	78.80	7	14.99
221	1	1	12	133	1	61.50	6	15.11
222	1	1	12	120	1	68.20	6	14.08
223	1	0	10	96	1	101.40	6	13.67
224	1	1	11	36	1	69.50	7	14.18
225	1	0	12	108				
226	1	1	12	12	1	27.60	8	14.69
227	1	1	12	28	1	71.80	7	15.35
228	1	0	11	78	1	71.80	6	14.46
229	1	1	12	64	1	68.10	6	13.89
230	1	1	11	76	1	35.60	6	14.22
231	1	0	10	129				
232	1	1	11	120				
233	1	0	10	136				
234	1	1	12	30	1	49.70	8	14.42

235	1	1	11	76				
236	1	1	12	68	5			
237	1	1	10	10	3			
238	1	1	11	93	1	45.50	6	13.05
239	1	0	12	24				
240	1	0	11	48	0			
241	1	1	12	39	1	45.30	6	14.26
242	1	1	10	46	1	70.80	6	14.29
243	1	0	12	16				
244	1	1	11	15	1	48.80	7	13.17
245	1	1	11	64	1	26.70	6	13.70
246	1	0	12	72	4			
247	1	1	11	100				
248	1	0	11	86	1	119.00	6	14.12
249	1	0	10	156	1	51.90	6	15.44
250	1	0	11	127				
251	1	0	11	28				
252	1	0	12	52				
253	1	0	12	27	1	56.80	6	12.08
254	1	1	12	52	1	112.00	7	15.85
255	1	1	12	16	1	61.30	6	15.37
256	1	1	10	123				
257	1	0	12	13	1	94.90	7	15.22
258	1	1	12	71	1	41.60	7	14.79
259	1	1	11	36	1	91.10	7	16.44
260	1	0	12	30	1	36.50	7	15.08
261	1	0	10	48				
262	1	1	11	64	1	91.30	7	15.33
263	1	0	12	54	2			
264	1	0	12	88	1	128.60	6	16.15
265	1	1	12	27	1	47.50	6	15.22
266	1	1	10	66	1	89.70	4	19.40
267	1	0	12	12	1	65.90	5	18.27
268	1	0	12	79	1	53.70	7	14.96
269	1	0	12	75	3			
270	1	0	12	40	1	94.20	6	15.24
271	1	1	12	123	2			
272	1	1	12	30	1	65.80	7	17.20
273	1	0	12	134	3			
274	1	1	11	108	1	24.10	5	14.57
275	1	0	12	150	1	95.90	9	13.71
276	1	1	12	112				
277	1	1	12	76	1	45.30	6	12.32
278	1	0	12	18	1	55.20	7	15.96
279	1	0	12	144				
280	1	0	12	125				
281	1	1	11	108	1	82.40	4	13.83

282	1	1	10	14				
283	1	1	12	96	4			
284	1	1	11	101	1	47.10	5	13.16
285	1	1	12	37	4			
286	1	1	10	15	5			
287	1	1	12	50	1	50.30	7	13.92
288	1	0	12	51	1	40.00	6	15.32
289	1	1	12	168	1	46.40	6	13.37
290	1	0	12	64	1	84.30	6	15.50
291	1	1	12	28	1	24.70	7	16.00
292	1	1	11	30	1	78.80	6	12.57
293	1	0	12	96	1	95.70	6	14.79
294	1	1	10	62				
295	1	1	12	112	1	141.00	7	15.91
296	1	1	11	12	1	63.30	7	13.75
297	1	0	12	118	3			
298	1	0	12	30	1	91.90	5	13.10
299	1	0	11	27	3			
300	1	0	12	160	1	91.80	5	14.63
301	1	1	10	122	1	36.90	7	15.89
302	1	1	12	120	2			
303	1	0	12	120	1	79.60	6	16.06
304	1	1	12	120	1	38.30	6	14.88
305	1	0	12	14	1	78.80	7	15.82
306	1	1	11	112	1	82.80	7	15.45
307	1	0	12	24	1	57.50	5	14.56
308	1	1	12	48	1	40.70	6	14.43
309	1	1	11	96				
310	1	0	12	52	1	69.30	6	13.53
311	1	1	11	120	1	88.00	5	13.76
312	1	1	12	108	1	40.30	6	14.44
313	1	1	10	30				
314	1	1	11	31	2			
315	1	1	10	54				
316	1	0	12	120				
317	1	1	11	12				
318	1	0	11	10	3			
319	1	1	11	102				
320	1	1	11	132				
321	1	1	11	125				
322	1	1	12	87				
323	1	0	12	123				
324	1	0	12	64	2			
325	1	1	12	180				
326	1	1	12	25				
327	1	0	11	64	1	107.40	8	15.49
328	1	0	12	186	1	67.90	8	15.65

329	1	1	12	102	1	69.20	6	14.97
330	1	1	12	125				
331	1	1	12	25				
332	1	0	12	108				
333	1	1	11	75	1	84.90	7	15.28
334	1	1	12	54	1	94.40	4	12.89
335	1	1	11	39				
336	1	0	11	84				
337	1	0	12	100	1	125.80	7	14.59
338	1	1	11	51	1	55.40	4	13.43
339	1	1	11	144	1	68.10	6	15.27
340	1	0	12	52	1	121.90	7	15.16
341	1	1	12	64				
342	1	0	12	147				
343	1	1	12	76	1	41.80	6	15.54
344	1	1	10	127	1	68.50	6	13.99
345	1	1	11	50	1	127.70	4	13.52
346	1	1	10	15	1	64.20	6	17.35
347	1	1	12	28	3			
348	1	0	12	195	1	87.20	7	13.03
349	1	0	12	120	1	109.10	5	16.32
350	1	1	12	185				
351	1	1	12	89	3			
352	1	1	12	136				
353	1	1	12	64				
354	1	0	12	51				
355	1	0	12	42				
356	1	1	12	120	1	35.10	6	17.32
357	1	0	12	32				
358	1	1	11	90				
359	1	0	11	45				
360	1	0	12	122				
361	1	1	12	120	1	117.70	7	14.62
362	1	0	12	84				
363	1	0	12	148				
364	1	0	12	64				
365	1	0	12	182	3			
366	1	1	11	188	1	44.90	6	15.26
367	1	1	12	16				
368	1	0	12	51	1	100.60	6	14.88
369	1	0	12	64	1	114.40	6	16.73
370	1	0	12	100	1	156.10	5	14.17
371	1	1	10	64	1	115.20	5	16.57
372	1	1	12	120	1	56.40	5	15.45
373	1	1	12	28	1	68.20	7	14.91
374	1	1	12	6	1	61.40	5	12.61
375	1	1	11	88				

376	1	1	12	126				
377	1	1	12	126				
378	1	0	12	63	4			
379	1	1	12	37				
380	1	1	12	108	3			
381	1	0	12	148	1	50.80	7	15.61
382	1	1	12	96	1	57.30	6	17.02
383	1	1	12	84	1	83.70	5	15.35
384	1	1	12	60	1	37.10	8	15.98
385	1	0	12	5	1	55.90	5	12.04
386	1	1	12	52	3			
387	1	1	11	5	3			
388	1	1	12	56				
389	1	1	12	75				
390	1	0	12	120				
391	1	1	12	112				
392	1	0	12	28	1	75.50	6	14.15
393	1	0	12	75				
394	1	1	11	66				
395	1	1	10	27	1	88.70	6	13.89
396	1	1	11	48	1	80.70	6	14.87
397	1	1	12	244				
398	1	1	11	66	0			
399	1	1	12	127				
400	1	1	12	123	0			

SUPPORT	RULES	BEDROOM MEDIA	PERSONAL MEDIA	PLAY EQUIP.	RESIDENTIAL DENSITY
2.25	15	3	3	6	1
3.75	14	0	0	8	3
2.25	13	0	0	3	2
2.25	9	0	0	5	2
2.25	4	0	0	5	6
2.75	15	1	1	4	2
2	14	0	0	2	2
2	14	0	1	4	2
2.5	12	1	1	7	1
3	15	0	2	5	2
2.5	14	0	1	9	2
2.75	15	0	2	4	1
4.25	14	0	2	3	3
3.75	14	0	1	6	2
1.75	14	1	1	5	2
3.75	14	0	1	7	2
1.75	14	0	3	5	2
3	15	2	2	7	1
1	15	0	0	6	1
2.5	15	0	1	3	2
4.5	15	4	2	5	3
3.5	11	0	2	9	2
2.5	14	0	2	2	2
1.5	14	0	2	3	1
2.75	13	0	1	5	2
4.5	14	0	1	11	3
2	13	0	1	1	1
2	6	0	1	4	2
1.5	14	2	2	2	1
2.75	14	0	1	6	1
2	13	0	0	4	2
2.25	9	0	0	4	1
5	12	0	1	6	3
4.25	14	1	2	9	2
2	13	0	2	6	1
2	15	1	2	4	2
4	8	2	1	3	2
1.75	15	1	0	5	4
2	15	3	1	6	2
2	10	0	0	4	1
2.5	8	0	0	3	3
1.75	14	1	0	3	3
2	12	0	0	0	2
4.25	12	0	1	9	2
2	13	0	0	6	1
2.25	13	0	0	1	1

2	15	0	2	6	6
2.75	14	1	2	5	2
4	14	0	0	7	2
3.25	13	0	0	9	2
2.25	14	0	1	4	3
3.25	9	0	0	3	2
1.75	11	0	0	1	1
2.25	13	0	2	5	1
1.75	7	0	1	5	2
4.5	13	0	0	2	1
2.5	14	0	0	2	2
3	14	0	0	2	3
2	15	0	0	2	3
1.75	14	0	1	7	2
1.25	15	0	0	0	4
2	11	3	2	6	2
2	14	2	2	3	1
3.5	14	0	1	5	6
2.75	12	3	1	13	1
4.5	13	1	0	4	1
1.25	14	2	0	4	2
2.5	14	0	0	5	2
4.25	15	0	1	4	4
2	15	1	1	3	1
4.25	15	0	0	6	2
1.25	14	0	1	1	6
3.25	15	0	0	2	1
2.25	15	0	0	5	2
2	13	0	0	4	4
1.75	15	0	1	7	3
3	11	0	1	5	1
4	15	0	0	1	2
1	15	0	0	2	6
1.75	10	1	1	4	2
3	14	0	2	5	1
1.75	15	1	1	6	2
3	14	0	2	5	1
3.75	13	0	2	8	4
1	14	0	0	1	2
2	13	0	0	3	2
3	13	0	1	4	2
3.5	13	0	0	5	3
2.75	15	0	0	4	2
2.75	12	1	1	5	3
1	14	0	2	4	2
2	13	0	0	5	2
1.25	11	0	0	4	3

2	15	0	0	7	3
2	13	0	0	0	5
2.75	15	2	1	6	1
3.25	13	0	0	4	2
5	12	3	0	4	1
4	13	0	0	7	1
3	13	0	0	4	6
4	15	0	2	6	5
1.5	12	3	2	4	3
3.25	13	0	0	4	2
3.5	15	0	0	4	3
2.25	12	0	1	4	4
3	13	0	0	7	2
2	14	0	0	2	1
2.75	13	2	0	10	2
2.75	13	0	0	3	1
3.5	14	0	0	4	1
1.5	15	1	1	4	3
3	12	1	1	10	2
2	11	1	0	2	2
1.75	14	2	2	5	2
3.25	14	4	1	2	3
2.25	14	0	1	8	3
2.25	14	0	1	4	1
1.75	12	0	0	2	2
2.25	14	0	0	4	1
3	14	0	1	6	3
3.75	12	1	1	4	1
3.5	14	1	1	5	3
2.25	10	2	0	10	4
2.75	13	4	3	7	1
3.75	11	0	0	5	2
2	13	0	0	1	3
3.25	14	0	1	7	1
2.75	13	0	1	4	1
3.25	13	0	0	5	2
3	6	1	0	3	1
2.75	8	3	3	10	2
2.5	9	0	0	3	3
2.25	13	0	0	4	3
1	14	1	0	7	4
1.5	14	0	0	2	1
4.25	14	3	2	7	3
3.25	13	0	0	5	3
2	15	0	0	4	2
3	11	0	0	11	1
2	14	1	2	4	6

3	12	0	0	3	3
3.5	15	0	1	8	2
2.25	14	0	3	5	2
2	12	2	1	11	2
2	15	1	1	4	1
2.25	10	1	0	2	1
2.75	12	0	1	6	2
1.75	14	0	0	3	1
4.75	14	0	0	8	2
2.25	13	0	0	5	2
1.5	13	0	0	10	2
3	12	0	2	1	1
1.75	15	0	0	5	2
2	15	0	0	6	2
3.25	14	0	1	7	4
4	11	0	0	4	1
3.5	13	0	0	2	3
2.75	14	1	0	4	2
3.25	11	1	1	6	4
4.5	15	0	0	3	3
2.5	13	0	0	2	6
2.5	13	0	2	2	2
2.25	13	0	0	4	4
2.75	15	0	1	7	4
1.75	15	0	0	4	2
2	13	0	0	1	4
2.5	15	0	1	3	2
4	13	0	0	7	6
2.25	14	0	2	5	4
3	9	4	1	7	6
2	15	0	0	1	2
2.75	10	2	0	3	3
4	15	4	1	7	3
1.5	14	0	0	3	1
4	15	0	0	7	3
2	15	2	1	5	2
3.5	15	0	1	4	3
4.25	14	0	0	3	4
3	14	1	0	5	4
4	15	0	0	4	1
1.75	9	1	1	8	4
3.5	12	1	1	6	2
2	13	0	0	4	2
3.5	14	0	0	4	2
2.25	13	4	3	4	3
3.5	15	0	0	1	2
2.75	13	1	0	4	3

2	14	0	0	3	2
5	12	1	1	7	2
1.5	15	0	1	6	2
3	14	0	0	4	1
1.5	13	0	0	9	4
3	14	0	1	5	2
3	12	1	0	6	3
3.25	9	0	0	4	4
4.5	14	0	0	1	2
3	15	1	1	8	2
3	12	0	0	0	6
2.75	14	4	3	12	3
2.5	15	0	0	7	1
3	13	0	1	5	2
2.5	13	0	1	8	2
2.5	14	0	0	4	3
4	14	1	0	2	1
1.5	13	0	0	0	1
4.5	13	0	0	3	2
2.75	13	0	0	4	2
2.25	14	0	0	2	1
1.75	13	0	0	1	6
2	11	2	0	3	4
4	14	0	0	0	2
2.25	12	0	0	3	1
1.75	12	0	0	3	6
2	11	1	3	10	2
3.25	15	0	0	5	1
2	14	0	0	4	1
2.25	14	0	0	5	1
2.5	9	0	0	1	1
3.5	13	0	0	10	3
2	13	4	3	7	2
3	15	0	0	6	1
2.75	11	1	0	5	6
3	14	0	0	2	6
1.25	15	2	0	2	2
2.25	15	2	0	1	6
2.5	14	2	1	3	3
4.75	13	3	0	3	2
2	13	2	2	5	1
3.75	14	0	0	3	3
1.25	14	0	0	2	6
1.75	12	2	0	1	6
2.25	11	0	0	4	1
1.5	15	0	0	6	1
2	15	0	0	7	1

1.5	13	0	0	5	4
3	11	0	0	2	2
3.5	13	0	0	2	6
3	14	1	1	5	2
1.25	13	0	0	2	6
2.5	13	0	0	2	2
1.5	15	0	0	3	6
2.5	12	1	0	2	3
2.5	10	0	0	3	6
3.5	13	2	2	5	3
4.5	13	0	0	0	4
2.75	10	1	0	2	6
1.75	15	0	0	4	1
2	15	0	1	2	2
1.75	12	0	0	1	6
1	12	0	0	0	2
3.25	9	0	0	1	6
2	14	0	0	3	6
2.75	14	0	0	2	3
1.25	12	0	0	0	3
4.25	15	0	0	1	6
1.5	11	0	1	3	2
3.5	10	0	1	2	6
1	14	0	0	0	1
2	14	0	1	3	6
1.25	13	0	1	2	6
2.5	11	0	0	1	1
2.5	13	0	0	2	6
3.75	14	0	0	4	3
2	12	0	1	1	3
3.75	13	0	0	2	6
1.75	14	0	0	2	3
3.75	13	0	1	4	4
3	11	0	0	2	4
1	13	0	0	1	3
1.75	9	1	1	0	6
3.25	15	0	0	4	1
1	11	0	2	1	6
1.75	13	0	0	3	6
4	9	0	1	4	3
5	13	0	0	2	5
3.5	9	1	0	3	6
3	13	0	0	3	1
3.5	13	0	0	2	1
2.5	11	0	0	0	6
1.5	11	0	1	1	3
4	12	1	0	1	1

4	13	0	0	1	2
2	11	0	0	3	6
1	8	0	0	2	6
3.5	14	0	0	3	1
1	15	0	0	3	5
4	14	2	0	1	2
2.25	13	1	1	6	6
4.25	15	0	0	4	2
1	13	0	0	2	1
2	15	0	0	0	6
4.25	15	0	0	2	3
1.5	15	1	0	2	1
1.75	12	0	0	3	6
1.75	13	1	1	3	3
3	10	1	0	3	6
4	7	0	0	1	1
2.25	13	0	0	1	1
2.25	14	0	0	2	1
4.75	13	0	0	6	6
2.75	14	0	0	1	6
1.75	15	0	0	2	2
1.25	14	0	0	2	2
2.25	8	2	3	5	3
1.75	15	0	0	3	1
3.5	15	0	0	1	1
3.5	12	0	0	4	6
1	10	0	0	1	6
2.25	8	0	0	3	6
2	14	0	0	1	4
1.25	12	0	0	0	6
2	12	0	0	2	2
2	13	0	0	1	1
1.25	13	3	3	0	6
2.25	11	0	0	2	6
2.5	14	1	1	6	3
3.5	14	3	1	6	6
4.25	13	0	0	1	6
2.25	13	0	0	2	1
1.25	12	1	3	0	1
4.75	12	1	0	3	5
2	9	0	0	4	6
2	9	0	1	1	6
2.75	15	0	0	3	3
2.25	11	0	2	4	3
2	13	0	0	4	6
4.25	13	0	1	2	3
1	12	0	0	1	4

3.5	15	0	1	4	3
1	13	0	0	3	6
2.25	14	2	2	3	4
3	15	0	0	1	1
2.75	14	0	0	3	6
1.5	11	0	0	3	6
4.25	14	2	0	5	5
1.75	12	1	0	4	3
3.5	12	1	0	3	4
3.25	15	0	0	7	3
3	14	1	1	2	6
2.75	15	3	0	1	2
3	14	0	0	1	6
1.75	14	0	0	0	6
3.25	9	2	1	4	6
1	14	1	2	1	6
1	9	0	0	1	6
2	12	0	0	0	6
2.5	14	0	0	1	2
1	13	0	0	3	6
1.75	12	0	0	1	6
1.75	13	0	0	0	1
2.75	14	1	1	3	6
2.5	13	0	1	1	6
1.25	8	0	0	0	2
2.5	11	2	0	3	6
3.5	13	0	0	0	6
1.25	13	0	0	2	6
1.5	11	2	1	1	6
2.75	12	0	0	3	6
2.25	14	1	0	2	6
2.25	13	0	0	0	6
2	12	0	0	0	3
3	13	0	0	4	6
2.75	12	0	0	1	6
4	14	0	1	3	1
1.25	14	0	0	1	6
1	12	0	0	1	6
1.75	10	0	0	3	3
2.5	11	2	0	4	6
2.75	10	1	2	6	3
3.25	14	0	0	4	1
1	13	0	0	0	6
3	10	0	0	0	6
1.25	13	0	0	1	6
3	14	0	0	0	6
4.25	15	0	1	2	3

1.5	14	1	1	1	6
2.5	13	2	0	2	6
1.75	10	1	1	4	3
2	10	0	0	3	5
4	14	0	0	0	6
3.25	14	0	0	3	2
4	7	0	0	4	4
4.25	7	1	0	4	1
5	15	4	2	9	1
4.25	13	0	0	2	3
3.75	12	2	1	5	4
3	11	0	0	0	6
1.5	13	0	0	0	6
2.25	12	0	0	2	6
3	13	0	0	2	1
3.25	14	0	0	3	2
2.5	13	0	0	1	3
2.25	12	0	0	2	6
3	8	0	0	2	6
1	11	2	2	4	6
3.25	9	0	0	2	6
5	9	2	1	4	1
2	7	0	0	2	6
1	14	1	1	2	6
3	15	2	2	3	6

LAND USE (DESTINATIONS)	LAND USE (RECREATION)	LAND USE ACESS	STREET CONNECTIVITY	SIDE WALKS
2.95	2.00	2.57	2.80	2.80
3.19	3.75	2.71	3.00	2.40
1.76	2.00	2.71	2.80	1.00
1.81	1.00	2.71	2.00	1.80
3.10	2.50	3.43	3.00	1.00
2.76	2.50	3.86	3.80	1.60
2.19	1.50	3.29	2.80	1.80
2.00	1.50	2.57	2.80	2.00
3.86	1.75	3.71	2.40	1.60
2.33	1.50	3.43	3.00	1.60
3.14	2.50	3.29	2.80	1.60
1.67	1.00	2.57	2.80	2.00
3.24	2.00	3.00	2.80	1.20
2.00	1.00	3.57	1.80	1.60
3.67	2.75	3.71	2.80	2.00
1.86	2.50	3.00	1.80	1.80
1.90	1.00	2.57	2.60	2.20
2.52	1.50	2.71	2.40	1.80
2.24	1.00	2.43	2.00	1.60
3.90	3.75	3.14	3.40	2.20
2.33	3.00	2.14	2.20	2.00
2.19	1.75	3.00	3.60	2.60
3.33	3.00	3.57	3.40	2.40
3.24	2.00	2.71	3.00	2.20
3.52	1.00	2.00	2.80	2.40
4.14	3.00	4.00	3.40	3.20
2.29	1.00	2.71	2.60	1.60
2.62	1.25	1.71	3.40	1.00
2.24	1.25	2.57	3.40	2.60
3.81	3.50	4.00	3.60	1.40
2.10	1.50	3.14	2.80	1.60
2.57	3.00	2.57	2.60	1.60
1.81	1.00	3.29	3.40	1.60
2.24	1.00	2.14	3.00	2.40
2.38	1.00	2.57	2.60	2.20
2.67	1.25	3.43	3.80	1.80
2.10	1.75	2.86	2.60	3.20
4.76	4.25	1.00	3.20	3.40
3.38	1.00	3.00	3.40	1.60
1.38	1.25	2.00	1.80	2.20
2.95	1.00	3.57	3.00	2.60
2.52	1.25	2.86	2.20	2.60
2.48	1.75	2.57	2.60	1.60
3.90	1.75	3.00	3.60	2.40
1.62	2.00	2.57	3.40	2.20
2.38	1.00	2.43	2.20	2.20

3.38	1.00	2.71	3.60	3.00
2.86	2.25	3.29	2.20	1.40
2.81	2.25	3.00	3.20	2.40
3.14	3.75	2.43	2.40	1.80
2.38	1.50	2.57	2.80	1.60
2.76	1.75	3.43	2.40	2.20
3.52	3.50	4.00	3.00	2.80
1.76	1.00	2.14	2.40	1.60
1.90	1.50	2.29	3.20	1.00
2.43	2.25	3.00	2.80	2.40
3.24	1.25	3.86	3.00	2.80
2.48	2.50	3.71	3.20	3.60
2.19	1.00	1.71	3.40	1.60
1.95	1.00	3.43	3.40	2.20
4.14	4.25	3.00	1.80	2.80
2.38	1.50	2.86	3.20	3.40
2.33	1.75	2.14	2.80	2.00
3.38	2.00	3.14	3.60	1.40
2.14	2.75	3.00	2.40	3.40
2.38	1.00	3.71	3.60	2.60
2.67	2.75	2.86	2.80	2.20
2.19	1.75	2.86	2.80	2.60
4.81	4.00	4.00	3.60	3.40
2.86	1.50	2.86	2.60	1.40
3.43	2.00	1.57	3.20	2.40
2.48	1.00	3.86	3.40	3.20
3.14	1.00	3.29	1.60	2.20
3.14	3.25	3.86	3.00	2.20
2.81	2.25	2.43	3.40	1.80
3.10	3.75	4.00	3.60	2.80
2.95	1.50	3.00	2.80	2.80
3.29	3.00	3.29	2.80	3.00
2.24	1.50	2.29	3.40	1.60
1.76	1.00	1.14	1.60	2.20
3.14	1.75	2.71	3.40	4.00
2.90	3.50	2.86	2.40	2.40
3.10	1.75	2.71	3.20	4.00
3.10	3.00	3.86	2.60	3.00
3.33	1.00	3.00	2.40	1.60
3.43	1.50	3.43	2.40	1.40
2.24	2.50	3.57	2.80	1.00
4.86	4.50	2.00	2.40	2.80
3.19	1.00	3.43	3.20	1.60
2.95	2.00	2.86	2.60	2.20
2.14	1.50	2.29	2.60	2.00
3.86	3.00	2.43	3.40	1.40
3.67	3.75	3.86	2.60	2.00

3.05	1.50	2.43	2.60	2.20
3.57	1.00	3.86	3.40	4.00
2.62	3.50	3.43	2.80	3.00
2.67	2.50	3.14	2.20	1.60
2.14	2.25	1.29	1.60	1.80
3.57	2.00	3.43	3.40	3.20
3.10	2.00	3.00	3.20	1.00
3.24	1.75	2.71	1.40	1.00
3.52	1.00	2.71	4.00	4.00
3.05	1.00	2.43	2.80	1.80
2.81	3.25	3.14	2.40	3.00
2.90	3.00	2.57	3.20	3.20
4.24	2.50	3.43	3.60	2.80
1.90	1.00	2.29	3.00	1.80
2.57	2.50	1.43	3.00	1.80
1.52	1.00	3.29	2.20	2.00
1.76	1.50	1.43	3.00	2.80
2.35	1.50	2.57	3.00	2.80
2.19	2.50	2.71	2.40	2.40
4.29	4.00	3.00	2.60	2.00
2.90	3.25	3.43	2.80	2.60
1.48	1.00	1.43	1.20	1.00
2.90	1.50	3.29	3.40	1.40
3.71	3.25	3.71	3.60	1.80
3.00	2.00	2.71	2.60	2.20
2.48	1.00	2.57	3.00	1.20
1.48	1.00	3.00	3.00	1.60
2.71	1.50	2.29	3.20	1.40
3.71	3.00	4.00	3.40	2.40
2.62	2.25	1.57	1.40	2.20
2.81	4.00	2.57	2.80	2.60
2.90	1.75	3.29	3.00	1.60
2.76	2.50	3.57	3.20	2.80
3.00	2.00	2.71	3.40	2.00
2.95	1.25	2.00	2.40	2.40
2.62	1.00	3.43	2.60	2.40
2.86	1.50	2.57	3.80	1.60
2.95	2.50	2.57	2.60	2.60
1.62	1.00	3.29	1.80	2.60
3.00	2.50	3.57	2.60	2.60
3.05	1.00	3.57	3.80	3.20
4.00	2.50	3.71	4.00	1.60
2.14	3.00	3.14	4.00	3.40
2.67	1.00	3.00	2.60	2.80
2.05	1.00	2.14	3.40	2.00
4.29	2.25	3.43	3.80	2.60
2.62	1.00	2.57	2.60	1.80

3.86	1.00	3.14	2.80	1.00
2.52	1.00	2.29	3.40	1.60
2.19	1.75	2.57	3.20	1.20
1.57	1.50	2.43	2.40	2.60
3.81	2.00	3.29	3.20	2.60
1.86	1.00	2.43	2.20	2.00
3.62	5.00	3.43	2.40	2.40
2.19	2.25	1.14	2.00	1.60
3.05	2.50	1.29	2.80	2.40
1.86	1.00	2.71	2.60	1.80
3.81	3.00	3.14	3.00	1.80
2.33	1.00	2.29	2.20	2.80
3.52	2.00	2.43	2.80	2.20
3.38	1.75	1.43	3.40	2.80
3.29	1.25	2.14	3.80	2.20
1.86	3.00	2.71	2.80	2.20
2.05	1.00	2.29	2.40	1.40
3.67	1.00	2.29	2.80	2.80
2.48	2.75	2.43	3.00	2.40
3.14	1.00	2.57	3.20	3.00
2.90	1.00	1.86	2.60	2.00
1.76	1.00	2.29	1.80	1.20
2.33	1.75	2.43	3.00	3.80
3.71	4.50	2.71	3.20	2.00
2.43	1.50	2.14	2.20	4.00
2.43	1.75	2.43	2.20	2.40
3.67	1.00	2.29	2.40	1.60
3.00	1.25	2.43	2.80	3.40
3.24	2.75	2.71	3.00	2.20
2.95	1.00	2.71	3.60	3.40
1.81	2.50	3.14	2.20	2.40
2.33	2.00	3.00	2.60	2.00
3.14	3.00	2.71	2.00	2.00
2.14	1.50	2.57	2.20	2.60
2.90	1.75	2.86	2.40	2.40
2.67	1.25	2.43	2.60	2.20
2.05	1.00	3.43	2.80	1.80
2.52	2.50	2.71	3.00	2.80
2.10	4.00	3.14	1.60	2.60
2.00	3.75	2.43	2.00	3.60
1.86	2.50	3.29	2.00	3.40
2.10	1.25	3.43	3.00	2.80
1.86	1.00	2.71	3.20	2.20
2.62	1.50	3.43	2.60	2.20
1.81	2.25	3.14	3.20	2.80
3.05	1.00	3.57	3.20	3.00
2.43	2.00	3.43	2.60	2.60

2.29	1.25	2.14	3.00	3.20
2.48	1.25	2.71	3.40	3.40
2.62	1.00	3.43	3.00	2.40
4.00	4.75	2.86	2.40	3.80
2.38	3.25	3.14	2.60	3.00
2.33	1.25	2.71	3.20	3.40
1.57	3.50	1.86	2.00	2.20
2.14	2.00	3.00	3.20	3.00
2.10	1.00	2.71	2.00	2.00
2.52	1.75	2.57	4.00	1.60
2.52	2.00	3.57	2.20	1.60
1.52	1.00	3.43	2.80	2.40
2.29	1.50	2.71	2.80	2.20
2.29	1.25	3.43	3.40	2.00
2.52	2.00	2.43	3.80	2.80
3.43	1.00	2.86	2.60	3.00
2.29	2.75	1.43	2.00	2.60
1.33	1.00	2.14	2.80	1.40
2.90	1.00	3.57	3.00	1.40
3.48	1.00	3.29	3.20	1.60
2.52	1.00	2.29	3.20	2.60
4.71	2.75	2.43	3.20	2.00
2.57	3.50	1.71	2.00	1.60
2.05	1.25	3.29	2.00	2.40
3.14	1.50	3.57	2.80	2.00
3.24	2.25	3.14	3.40	2.40
3.95	4.50	3.86	2.60	2.40
3.00	1.00	2.71	3.00	1.60
1.90	1.25	3.29	3.40	1.20
4.19	3.25	4.00	3.20	2.80
2.19	2.75	3.43	2.80	1.60
1.81	1.00	3.00	2.60	2.00
1.62	1.00	3.57	3.40	1.00
3.48	3.50	2.86	3.60	2.00
4.43	4.00	1.86	2.40	2.60
2.38	3.50	2.86	3.40	1.00
3.10	2.50	2.71	3.40	1.80
2.33	1.00	3.14	3.40	2.60
2.19	1.50	2.57	2.00	2.40
3.00	1.50	3.14	2.60	1.80
2.76	4.25	3.57	2.60	3.60
3.24	2.75	4.00	3.40	2.60
2.81	1.25	2.86	3.60	1.80
2.90	1.00	3.00	3.00	1.40
1.90	2.25	1.71	3.40	3.20
2.38	1.75	2.43	2.00	2.80
1.24	1.00	3.00	2.20	2.40

1.57	1.75	2.86	3.40	2.60
3.81	1.00	2.29	1.60	3.60
3.48	1.00	3.43	3.60	1.00
2.10	2.50	2.57	2.40	3.20
2.62	3.50	3.43	3.40	2.20
3.19	3.00	3.29	3.80	2.20
2.14	1.00	3.14	3.00	2.20
2.62	1.50	2.71	2.60	3.20
1.48	1.75	3.43	3.40	2.00
4.43	3.50	3.00	3.40	2.60
3.14	1.75	2.14	3.00	3.40
2.95	3.25	4.00	3.20	2.00
3.14	1.00	4.00	3.60	2.60
3.19	1.75	3.00	3.00	1.60
3.48	2.50	3.29	2.60	1.00
2.38	2.25	1.57	1.80	1.60
2.43	1.25	3.00	2.80	1.60
2.33	1.00	1.71	2.40	2.00
1.95	2.00	1.71	2.40	2.80
3.33	2.00	2.43	3.00	1.40
2.43	1.00	3.14	4.00	1.60
2.10	1.00	3.00	3.60	2.60
2.00	3.50	2.71	2.60	3.20
1.76	1.50	4.00	3.40	1.60
3.43	2.75	3.71	3.40	1.60
2.57	3.00	2.43	2.40	1.80
4.05	3.00	3.86	3.60	2.20
2.10	1.00	3.43	3.40	2.20
2.38	1.00	4.00	3.40	4.00
3.43	2.75	3.57	3.60	2.20
3.14	1.00	3.14	3.40	2.80
3.67	2.00	2.29	3.00	1.60
2.29	1.25	2.29	2.80	3.20
2.81	3.50	3.00	2.20	4.00
2.33	1.50	3.00	2.80	2.20
2.57	1.00	2.14	3.00	1.40
3.14	1.25	2.71	1.80	2.00
3.05	1.00	3.00	2.20	2.20
2.38	2.00	3.14	3.60	2.20
2.90	1.75	2.86	3.40	1.80
1.76	1.00	3.86	3.60	3.20
2.38	1.75	1.71	1.40	2.60
2.10	1.00	3.43	2.20	2.80
2.19	1.00	2.43	1.80	1.80
1.38	1.00	1.86	1.60	3.20
3.24	3.25	3.14	2.00	2.00
3.81	1.00	2.86	3.20	3.60

1.48	1.00	2.71	3.40	2.20
2.48	2.00	3.14	3.40	2.60
2.81	1.00	2.29	2.20	2.00
3.62	1.50	3.29	3.20	3.00
2.90	1.00	3.86	3.20	2.60
1.52	1.00	3.43	3.20	2.80
2.71	3.00	3.29	2.40	3.00
4.05	3.75	3.43	3.40	3.00
1.67	3.00	2.29	2.80	2.60
3.29	2.00	3.00	3.60	1.80
1.95	1.00	3.14	3.60	2.80
3.00	2.50	4.00	3.40	2.20
2.52	1.00	3.29	3.40	2.40
2.29	1.00	2.14	2.20	1.40
2.52	2.00	3.00	2.80	2.60
2.86	3.25	1.57	2.20	1.60
2.19	1.25	2.57	3.00	2.80
3.90	1.25	3.29	3.80	2.60
2.14	2.25	3.57	3.40	3.00
2.76	2.25	2.86	2.00	2.00
2.57	1.00	3.57	3.60	2.40
2.62	1.00	3.57	3.20	2.60
2.33	3.50	3.29	3.60	2.20
1.38	1.00	2.86	3.80	1.80
2.24	1.00	2.14	2.80	2.20
2.24	2.00	3.00	2.40	2.00
1.33	1.00	2.14	1.80	1.80
1.86	1.00	2.29	2.80	2.80
1.90	1.00	3.00	3.20	2.20
1.95	1.00	2.14	1.60	1.60
3.29	2.25	3.71	3.80	2.00
3.10	1.25	2.57	3.20	1.60
2.29	1.00	3.00	2.80	4.00
4.24	3.75	3.43	3.40	2.00
3.48	2.00	4.00	3.40	2.00
2.38	1.00	2.71	2.20	2.20
4.10	3.00	1.57	3.40	1.80
2.48	1.00	3.43	3.00	3.80
2.24	1.00	1.57	2.00	3.60
2.52	2.25	2.43	3.00	2.40
3.81	3.50	3.57	3.40	1.40
2.57	1.00	2.29	1.40	1.80
3.67	1.75	3.14	3.00	1.20
3.81	4.00	3.43	3.60	3.20
1.81	1.00	2.71	1.60	2.00
3.38	2.25	3.43	3.40	2.20
1.71	2.50	2.29	4.00	3.20

4.05	3.25	3.00	3.40	1.00
2.33	1.25	2.57	2.40	2.20
2.24	1.00	2.86	1.80	2.00
1.86	1.00	2.00	1.60	2.40
3.81	2.75	3.29	3.20	2.40
1.81	1.00	2.71	4.00	1.60
2.57	1.00	2.71	2.00	2.40
2.86	3.00	3.14	2.60	2.40
2.43	3.75	2.71	2.40	2.40
2.52	2.25	3.71	3.00	2.80
3.29	2.50	3.57	3.80	3.00
2.86	1.75	3.14	3.60	2.40
3.43	1.00	3.71	3.20	2.20
3.62	1.75	3.57	3.60	2.20
2.00	3.50	2.43	2.40	1.60
1.29	1.00	2.43	2.40	1.00
2.43	1.00	1.86	3.60	1.00
1.81	1.00	3.29	4.00	2.80
2.19	1.25	3.14	3.40	3.40
3.43	3.50	2.71	2.20	1.20
2.71	3.75	2.29	2.00	2.20
2.19	1.00	1.86	2.20	2.80
3.33	1.50	3.29	2.80	3.00
1.24	1.00	2.00	3.20	1.80
1.43	1.00	2.71	3.20	1.60
1.33	2.00	3.57	3.20	3.00
3.10	1.00	3.57	3.00	2.80
2.62	5.00	3.43	3.80	2.80
3.67	2.00	3.71	3.40	1.60
2.29	3.00	3.43	3.00	2.60
1.71	2.00	2.71	3.20	1.60
1.67	1.00	2.71	4.00	3.40
2.43	3.00	3.57	2.80	2.20
2.62	1.25	1.71	2.80	1.80
2.67	1.25	3.29	2.40	2.80
3.24	2.25	3.43	3.40	2.00
3.24	3.00	4.00	2.20	1.00
1.90	2.00	2.29	4.00	3.20
2.62	1.50	2.14	2.40	3.60
1.38	1.00	3.43	3.60	3.00
1.67	1.50	2.00	2.20	2.80
1.43	1.00	1.71	3.60	1.80
3.43	2.00	1.29	1.40	2.40
2.00	1.00	3.00	2.80	1.80
2.33	1.00	2.14	2.00	1.60
3.10	1.00	2.14	2.40	2.00
4.05	3.25	3.00	3.40	1.00

3.24	1.25	3.14	3.20	1.80
3.33	2.50	3.29	3.60	2.20
2.67	3.00	3.57	2.20	2.40
3.43	2.25	3.57	3.60	3.00
2.95	3.00	3.57	3.60	1.60
1.67	1.00	3.14	4.00	2.80
1.57	1.50	2.57	3.80	3.00
1.67	1.00	2.57	2.20	2.25
3.29	1.50	3.14	3.40	1.60
2.67	1.00	3.43	2.20	2.80
1.90	1.00	3.00	2.20	2.40
1.95	1.00	2.43	3.80	2.20
1.43	1.00	2.43	3.00	1.80
1.90	1.00	3.14	3.40	2.80
2.29	1.25	2.86	2.60	1.60
1.67	1.00	3.14	4.00	2.80
2.48	2.25	3.14	3.20	2.80
1.86	1.00	3.14	3.40	2.80
2.57	1.00	2.57	3.00	2.40
4.29	4.50	2.43	2.60	2.40
2.95	1.25	1.71	2.60	1.60
3.19	3.25	3.29	4.00	4.00
2.57	1.50	2.43	3.00	2.40
1.29	1.00	2.71	3.20	1.20
3.29	2.75	4.00	4.00	3.00

CROSSING	PATHS	WALK & CYCLE INFR.	AESTHETICS	TRAFFIC	CRIME	PERSONAL SAFETY	STRANGER DANGER
2.25	2.00	2.50	3.38	3.00	3.00	2.33	3.67
2.00	2.50	2.42	3.13	3.83	3.00	2.67	1.33
2.25	2.00	1.58	2.75	2.83	3.00	2.33	1.33
2.75	2.00	2.08	3.63	2.33	3.25	2.33	4.00
2.00	2.00	1.50	1.75	2.50	2.00	2.33	2.00
2.25	2.50	1.92	2.75	2.50	2.50	2.33	3.00
1.75	2.00	1.75	2.38	2.17	1.50	1.67	1.00
2.25	2.00	2.08	2.88	2.33	3.75	2.33	2.00
1.75	2.00	1.42	2.88	1.50	1.25	2.33	2.67
1.25	3.00	1.67	3.38	2.67	3.75	2.67	2.00
2.00	2.00	1.75	1.88	2.17	3.00	2.67	2.33
3.25	1.00	2.17	3.13	2.17	2.25	3.00	1.33
1.25	2.00	1.33	2.63	2.33	2.75	2.33	1.00
2.50	2.00	2.00	1.50	3.17	3.75	2.00	2.67
1.00	1.00	1.42	2.38	2.17	4.00	3.67	2.00
2.25	2.00	1.92	3.00	4.00	3.75	2.00	2.67
2.25	1.50	2.00	2.38	2.83	2.50	2.00	1.33
2.00	4.00	2.42	3.13	1.83	1.75	2.67	2.00
1.25	2.00	1.50	1.75	1.83	2.50	3.33	2.00
1.50	2.00	1.92	2.25	2.17	3.00	2.67	3.00
2.00	2.00	2.08	2.50	2.67	3.00	3.33	4.00
2.25	3.00	2.42	3.13	3.33	3.25	2.67	2.00
2.00	3.00	2.25	2.63	1.83	2.50	2.67	2.00
2.50	3.50	2.42	3.88	3.17	3.25	2.33	1.33
1.75	3.00	2.25	2.25	3.00	2.25	2.00	4.00
3.50	4.00	3.50	4.00	3.50	4.00	3.00	4.00
1.75	2.50	1.75	2.00	2.67	3.00	2.33	1.00
1.75	1.50	1.33	1.75	3.67	2.75	2.67	2.00
1.50	2.50	2.08	3.63	3.17	4.00	3.00	2.00
1.50	3.50	1.75	2.38	2.20	2.75	3.00	4.00
2.00	2.50	1.92	3.50	2.67	1.75	2.33	1.00
1.25	2.00	1.50	1.88	2.67	2.50	2.00	4.00
1.75	2.00	1.67	3.63	1.50	3.25	3.00	2.33
2.50	2.50	2.42	2.38	3.67	3.25	2.00	1.00
1.25	2.50	1.83	3.00	2.17	2.75	2.33	1.00
1.75	2.00	1.83	2.63	2.50	2.75	2.00	2.33
2.25	3.50	2.92	2.88	1.83	4.00	3.67	3.67
2.75	4.00	3.33	3.88	3.17	4.00	4.00	3.33
1.00	2.50	1.42	1.75	2.00	1.75	2.00	1.00
1.50	2.00	1.83	3.88	2.83	3.25	2.33	2.67
2.75	3.00	2.67	3.63	2.83	3.50	3.00	2.00
1.75	2.50	2.17	2.88	2.67	4.00	3.33	3.00
1.25	3.00	1.67	1.75	2.50	2.25	1.67	1.00
1.25	2.50	1.92	3.00	1.50	2.75	2.00	1.00
1.50	2.50	1.92	3.13	2.83	2.75	2.33	2.00
2.00	1.50	1.92	2.00	2.00	2.50	2.00	1.67

1.25	3.50	2.33	2.00	1.67	2.75	3.00	2.00
2.00	2.50	1.83	2.38	2.67	4.00	3.33	4.00
1.50	1.50	1.92	2.88	1.33	1.75	2.00	1.00
2.00	2.00	1.83	3.88	1.83	4.00	2.67	2.00
1.50	3.00	1.75	2.75	2.33	3.00	3.00	3.00
1.25	2.50	1.83	2.63	2.33	2.50	2.33	3.00
3.25	4.00	3.17	2.88	1.67	1.00	4.00	1.00
1.25	2.50	1.58	1.88	2.17	3.50	2.33	1.33
1.50	2.50	1.42	3.13	3.33	3.25	3.00	3.00
1.25	3.00	2.25	2.50	2.67	3.50	2.67	1.00
1.75	3.00	2.33	1.75	2.17	3.00	1.67	2.00
3.75	4.00	3.58	3.75	3.33	4.00	4.00	3.00
1.50	1.00	1.42	2.13	2.83	3.00	2.33	3.00
1.50	3.50	2.08	3.13	2.83	2.75	1.67	3.00
1.50	2.50	2.25	2.50	2.17	2.75	2.67	3.67
3.00	2.50	3.00	2.75	3.67	4.00	4.00	3.00
2.50	2.50	2.17	2.88	2.50	2.75	2.33	4.00
1.50	2.50	1.58	2.63	3.00	3.25	3.00	1.33
2.50	2.50	2.83	3.25	2.67	4.00	2.33	2.33
3.50	3.00	2.92	3.50	2.50	4.00	3.00	2.00
2.25	2.50	2.42	3.38	2.33	3.75	4.00	1.33
2.25	4.00	2.67	3.63	3.50	4.00	3.67	1.00
3.25	3.00	3.08	3.75	4.00	4.00	3.67	3.67
1.25	1.00	1.25	2.00	1.33	2.50	2.33	2.00
2.75	2.50	2.50	2.25	2.67	2.00	2.33	2.00
1.00	2.50	2.17	1.63	2.17	2.75	2.33	2.00
2.25	2.00	2.08	2.75	2.00	3.00	2.33	2.00
1.75	2.00	1.92	2.00	2.17	3.00	2.00	1.00
1.50	4.00	2.00	2.00	2.17	4.00	2.33	2.00
1.50	3.00	2.25	3.00	2.67	3.00	2.33	2.67
2.50	2.50	2.67	2.63	3.50	4.00	3.00	3.33
2.00	1.00	2.17	2.38	2.83	3.50	3.33	3.00
1.00	2.50	1.50	1.63	1.50	2.00	2.67	1.00
2.50	2.00	2.33	2.50	2.17	2.75	2.33	1.33
4.00	4.00	3.75	2.50	2.00	4.00	4.00	3.00
2.00	2.00	2.17	3.00	2.17	2.50	2.67	2.00
4.00	4.00	3.75	2.88	2.00	4.00	4.00	3.00
1.50	2.50	2.50	3.88	3.67	4.00	3.67	4.00
2.00	3.00	1.83	2.63	2.83	2.75	2.33	2.00
1.00	2.00	1.33	2.25	2.00	2.75	2.33	2.00
1.50	2.50	1.42	3.50	2.17	3.00	2.33	2.00
2.75	1.50	2.50	3.38	2.33	2.00	3.33	1.33
1.50	1.50	1.50	2.00	1.67	1.75	2.33	1.00
2.25	2.00	2.08	2.25	3.00	2.75	2.67	1.00
2.25	3.00	2.25	2.63	3.17	2.75	3.00	1.67
1.25	2.50	1.50	2.25	2.00	2.50	2.67	2.00
1.75	2.50	1.92	3.13	2.00	4.00	3.00	3.00

1.25	1.00	1.58	2.00	2.50	3.00	2.33	2.00
2.50	4.00	3.25	4.00	2.00	4.00	2.00	2.00
3.75	3.00	3.17	3.00	3.67	3.75	3.00	3.00
1.00	2.50	1.50	2.00	1.50	2.25	2.00	3.00
2.50	2.00	2.08	3.75	1.17	1.50	3.00	1.00
2.75	2.50	2.92	1.75	2.50	3.00	2.67	1.00
1.75	2.00	1.42	2.25	1.33	2.50	2.33	1.67
1.25	1.00	1.08	2.88	1.50	2.50	2.33	2.00
2.50	3.50	3.25	2.75	2.17	2.75	2.33	2.00
2.25	3.00	2.17	3.38	3.67	3.75	3.33	2.67
4.00	4.00	3.58	2.88	1.50	2.25	2.67	1.00
2.00	2.50	2.50	3.50	2.17	2.25	3.00	1.33
1.25	2.50	2.08	2.88	2.00	1.75	2.00	1.67
1.50	3.50	1.92	2.13	2.67	4.00	2.00	2.00
1.75	2.00	1.75	3.50	2.67	3.00	1.33	1.00
1.75	2.00	1.83	2.63	2.33	3.50	3.33	1.00
3.00	2.00	2.58	2.50	2.17	4.00	1.67	3.00
2.50	2.50	2.50	2.63	3.33	2.50	2.67	2.33
2.50	2.00	2.33	2.13	2.33	3.00	2.67	2.67
2.25	2.50	2.08	2.25	1.83	3.25	3.00	3.00
2.50	2.00	2.42	1.88	2.17	3.50	3.00	1.00
1.00	1.00	1.00	2.75	2.17	2.00	3.33	1.00
1.00	2.50	1.42	2.38	1.67	1.50	1.67	1.00
2.00	2.50	1.92	2.50	1.67	2.75	2.67	1.00
2.50	3.00	2.42	2.75	2.50	2.00	2.33	2.00
1.00	2.00	1.25	2.13	2.17	2.75	2.67	3.00
2.25	3.00	2.00	3.13	2.67	2.50	2.33	3.00
2.25	2.00	1.75	3.38	2.17	3.50	3.00	2.33
1.75	2.00	2.00	2.38	2.00	3.50	2.67	3.00
2.50	4.00	2.67	3.88	3.50	3.75	3.00	2.67
1.00	2.50	2.00	3.50	2.67	3.25	1.67	1.00
2.25	3.00	2.17	2.50	1.67	3.00	2.33	1.67
2.25	2.50	2.50	2.63	2.17	3.00	3.67	3.67
1.75	2.50	2.08	2.00	2.00	1.50	3.00	1.00
2.75	2.50	2.50	2.75	2.83	2.50	2.67	4.00
2.50	2.50	2.42	3.25	1.17	2.25	3.33	1.00
1.25	1.50	1.42	2.13	1.17	2.25	2.67	2.00
2.25	2.50	2.50	2.88	2.67	1.50	2.67	2.33
2.50	3.50	2.58	3.63	2.83	2.75	3.33	2.67
1.00	1.50	1.75	2.25	1.83	3.75	2.00	1.67
2.75	2.50	3.00	3.63	3.50	4.00	3.00	4.00
1.25	2.50	1.67	1.88	3.33	2.75	2.67	2.00
1.75	2.50	2.50	3.25	1.00	4.00	3.00	4.00
3.00	2.00	2.58	3.50	2.33	3.25	3.00	2.67
1.75	1.00	1.67	2.38	2.50	3.00	2.00	1.00
1.50	3.50	2.25	3.00	3.00	4.00	3.67	4.00
2.00	2.00	1.83	2.75	2.83	3.25	2.67	2.00

1.00	1.00	1.00	2.00	1.83	3.50	2.00	1.00
2.00	2.50	1.83	3.88	2.00	2.50	2.33	1.33
1.25	1.00	1.17	2.38	3.00	1.75	1.00	1.00
2.25	2.00	2.33	2.63	2.50	2.50	3.00	2.67
2.50	2.50	2.42	3.13	2.33	2.75	2.33	1.00
2.00	1.50	1.83	1.38	3.50	3.50	4.00	1.00
1.75	4.00	2.30	2.63	2.00	2.00	2.00	1.00
1.00	1.00	1.25	2.25	1.50	2.25	3.00	2.00
2.75	3.00	2.67	3.13	2.00	4.00	3.00	3.00
1.00	2.00	1.50	2.88	1.83	2.75	3.00	1.00
2.50	2.50	2.08	2.50	2.67	2.75	3.33	2.00
2.50	1.00	2.25	2.63	2.17	3.25	3.00	3.00
1.75	2.50	2.00	4.00	3.00	3.25	3.00	4.00
1.00	2.50	2.00	2.38	3.17	2.75	3.00	2.00
2.50	3.00	2.58	3.25	2.67	4.00	2.33	2.33
1.75	2.50	2.25	2.50	2.50	2.50	3.00	1.00
2.50	2.50	1.92	1.63	3.00	3.50	2.00	1.33
3.25	1.00	2.50	3.25	2.50	2.00	2.67	1.33
2.25	2.00	2.33	2.88	2.33	2.50	2.67	3.33
3.25	4.00	3.08	2.88	3.50	4.00	3.00	1.00
1.75	4.00	2.17	2.88	1.50	3.25	3.00	1.00
2.50	3.50	2.17	2.50	2.67	2.00	3.00	1.00
2.25	2.00	2.75	2.50	2.50	3.25	2.33	2.00
1.75	3.50	2.25	2.50	1.83	1.75	3.00	3.33
1.75	2.50	2.75	3.25	1.33	3.25	2.00	1.67
1.75	4.00	2.25	2.25	2.83	3.00	2.67	1.00
2.00	2.00	1.92	2.50	2.33	2.75	2.33	2.00
1.75	2.50	2.50	2.50	2.00	1.75	3.00	3.00
2.50	1.00	2.00	2.88	2.17	2.25	2.33	1.00
2.25	3.00	2.92	1.75	2.50	2.25	3.33	2.33
2.50	3.00	2.50	3.63	2.67	3.00	2.67	1.00
3.25	1.00	2.42	2.13	4.00	2.25	2.00	1.00
2.25	4.00	2.33	2.38	4.00	4.00	3.67	2.00
2.75	3.50	2.67	2.38	2.17	2.25	2.00	1.33
2.25	3.00	2.33	2.00	3.50	3.50	3.00	2.00
2.00	2.50	2.08	3.13	2.00	3.25	2.33	1.00
1.75	4.00	2.08	2.25	2.67	3.75	3.00	1.00
1.00	2.50	2.00	3.25	2.00	1.25	3.00	1.00
1.75	3.50	2.33	2.88	3.50	3.25	4.00	4.00
2.50	4.00	3.08	3.75	1.50	2.00	4.00	1.00
3.00	2.50	2.67	2.25	2.00	2.50	3.67	1.00
1.75	2.00	2.17	3.13	1.50	1.00	2.00	1.00
2.00	2.50	2.33	2.50	3.50	2.75	4.00	2.00
1.75	2.50	1.92	2.63	3.00	1.75	3.00	1.00
1.75	2.00	1.92	3.38	4.00	3.25	2.00	1.00
2.50	4.00	2.83	2.88	2.00	3.00	3.00	1.00
1.25	2.50	2.17	2.88	3.17	2.50	2.67	1.00

1.75	1.00	1.83	3.00	3.00	1.75	2.00	1.00
2.75	4.00	3.33	3.25	2.50	2.00	3.00	1.00
2.75	3.50	2.83	2.13	3.17	3.50	4.00	1.67
2.50	2.00	3.08	3.25	4.00	4.00	3.00	1.00
2.00	2.50	2.42	3.75	1.50	1.25	4.00	2.00
1.50	2.50	2.67	3.50	1.00	2.00	3.33	1.00
1.50	1.50	1.75	3.75	3.67	3.75	2.33	4.00
2.00	2.50	2.42	3.50	3.50	1.00	3.67	1.00
1.75	1.00	1.67	2.50	2.50	2.50	4.00	1.00
1.00	1.00	1.25	1.63	1.83	1.00	3.00	1.00
1.00	2.50	1.50	1.75	4.00	2.50	3.00	1.00
1.75	2.50	2.08	3.50	3.00	4.00	4.00	1.00
3.00	3.50	2.75	3.38	2.83	2.50	2.00	2.00
2.75	4.00	2.50	2.63	2.00	2.50	3.33	2.33
2.50	2.50	2.50	3.25	3.33	2.25	3.33	2.00
1.75	3.00	2.58	3.13	1.33	1.25	2.33	1.00
2.00	2.00	2.17	1.88	3.83	3.25	3.00	1.00
1.00	2.00	1.58	1.50	2.33	1.00	1.33	2.00
1.50	2.50	1.67	3.00	3.00	3.00	2.67	3.00
1.75	2.50	1.17	3.13	3.50	4.00	3.67	1.33
1.25	3.50	2.17	3.63	3.00	2.50	1.67	1.00
2.50	2.50	2.08	1.63	2.50	1.50	2.67	1.33
2.00	2.50	1.92	1.88	4.00	2.00	2.33	1.33
1.50	3.00	2.25	2.88	2.00	3.00	3.00	3.00
4.00	3.00	2.83	2.75	3.50	3.00	3.00	2.00
3.25	2.00	2.50	1.75	3.17	2.50	2.00	1.67
1.75	2.50	2.08	2.88	2.50	3.25	3.00	2.33
2.50	3.00	2.25	2.75	2.33	2.50	2.67	1.33
2.75	2.00	1.83	1.75	1.83	1.75	2.67	2.00
2.50	2.50	2.50	2.88	3.83	4.00	3.00	3.00
2.50	1.00	1.75	2.13	2.50	1.50	3.00	2.00
2.50	3.50	2.50	3.00	3.50	3.75	3.00	3.00
1.75	1.00	1.25	3.25	2.67	2.25	2.00	3.33
2.25	2.50	2.25	3.00	2.67	3.25	2.67	2.67
3.75	4.00	3.33	2.50	1.17	4.00	4.00	2.00
2.50	2.50	2.00	2.13	3.00	2.50	3.00	4.00
1.50	1.50	1.58	3.13	2.67	3.50	2.00	2.00
2.25	3.50	2.67	3.38	2.83	2.50	2.67	1.00
3.00	1.50	2.33	3.50	3.33	3.25	3.00	1.33
2.50	3.50	2.25	2.63	3.33	3.25	3.00	2.33
3.00	3.50	3.25	3.13	3.17	3.25	3.33	2.00
1.75	3.50	2.33	3.38	2.83	4.00	2.67	4.00
2.50	3.00	2.08	2.50	2.00	3.50	2.33	1.00
1.50	3.00	1.75	1.38	1.83	2.50	2.00	2.00
2.50	3.50	3.17	3.38	1.83	2.50	3.33	1.00
1.75	3.50	2.58	3.50	3.67	2.50	4.00	4.00
2.75	3.00	2.67	3.00	1.83	1.50	3.00	1.00

3.25	4.00	2.92	3.25	2.50	3.50	3.00	2.33
3.00	3.00	3.25	3.00	2.67	3.25	4.00	1.33
1.50	4.00	1.92	3.00	1.50	3.00	3.33	3.33
2.50	3.00	2.92	2.75	2.33	1.75	2.67	2.00
2.00	2.50	2.25	2.50	2.17	2.25	3.00	2.00
1.25	3.50	2.00	2.50	3.50	3.75	2.67	4.00
1.75	1.50	1.83	1.50	2.33	1.25	3.00	2.67
1.50	2.00	2.33	1.75	2.17	2.75	2.00	2.00
3.25	3.00	2.50	2.88	2.83	3.75	2.33	1.67
3.75	4.00	3.33	2.13	3.17	4.00	3.00	2.00
2.50	2.50	2.75	2.63	3.00	3.00	2.67	2.00
3.50	4.00	2.75	2.75	3.17	2.50	3.33	3.00
3.75	1.00	2.58	2.75	3.83	4.00	4.00	1.00
1.25	1.00	1.33	2.88	2.83	3.00	3.00	3.00
1.00	2.00	1.17	1.88	2.83	2.50	2.67	4.00
2.50	4.00	2.25	2.25	3.00	1.50	3.00	2.00
1.75	2.50	1.75	3.50	2.50	4.00	2.67	3.33
1.75	1.00	1.67	2.75	1.50	3.00	3.00	2.00
2.00	2.50	2.33	3.25	2.17	1.50	2.67	1.00
2.25	2.50	1.83	1.63	2.67	3.25	3.00	2.67
1.00	2.00	1.41	1.75	1.83	1.75	2.00	2.00
1.75	3.00	2.25	3.25	3.17	2.75	2.00	1.00
2.50	3.00	2.83	2.63	2.00	1.50	3.00	2.33
1.25	1.00	1.33	2.75	2.00	2.50	3.00	1.00
1.75	2.00	1.75	1.75	1.50	1.25	2.67	1.00
3.25	3.00	2.58	2.63	3.00	2.50	3.67	3.00
2.50	3.50	2.50	1.88	3.00	3.25	2.33	4.00
1.75	3.00	2.08	2.63	2.17	2.75	2.67	2.00
4.00	4.00	4.00	2.50	2.50	3.25	3.00	4.00
1.75	3.50	2.17	3.38	4.00	3.75	4.00	3.00
2.50	2.50	2.50	2.88	3.00	2.50	4.00	1.00
1.75	2.50	2.00	3.75	3.50	2.75	2.67	2.33
2.00	3.00	2.58	3.25	2.67	1.50	2.00	1.67
3.25	1.50	3.33	4.00	3.50	2.50	4.00	1.00
2.50	2.50	2.25	2.50	3.50	4.00	4.00	1.00
1.00	2.00	1.33	1.63	1.00	2.00	2.67	1.33
1.75	1.00	1.67	2.75	1.83	2.50	2.33	1.00
2.50	3.00	2.33	2.50	4.00	4.00	3.00	4.00
2.00	1.00	1.83	1.00	3.00	1.25	2.67	1.00
2.00	2.00	1.83	2.88	4.00	3.50	3.00	3.00
3.50	3.00	3.33	3.88	2.17	3.00	3.33	2.67
1.75	2.00	2.08	2.50	3.83	3.25	2.33	4.00
2.50	4.00	3.00	2.63	2.17	3.50	3.00	1.33
1.00	1.00	1.50	2.63	2.67	2.50	2.33	2.00
3.00	4.00	3.25	3.13	3.17	1.50	2.00	3.00
1.00	2.50	1.67	2.38	2.00	2.00	1.67	2.33
2.00	2.00	2.83	1.88	3.00	1.00	3.00	1.00

3.50	3.00	2.67	3.13	3.17	3.00	2.00	1.33
1.50	2.50	2.08	2.63	2.17	1.75	2.33	1.00
3.50	3.00	2.83	2.63	1.83	3.75	3.00	4.00
2.50	3.50	3.00	3.00	1.50	1.00	2.67	1.33
1.75	2.50	2.33	2.25	2.00	2.25	2.67	1.00
3.00	3.00	2.83	3.13	1.17	1.25	2.00	1.00
3.00	3.00	3.00	3.25	1.33	1.25	2.67	2.00
2.50	3.50	2.92	2.88	2.33	3.25	3.67	1.00
2.00	2.50	2.25	2.75	2.00	2.25	2.00	2.33
1.00	2.50	1.58	2.13	2.00	2.75	2.33	1.00
1.00	4.00	2.25	3.13	3.17	1.50	3.00	1.00
1.75	4.00	2.25	2.88	3.50	3.25	2.00	1.00
1.75	2.00	2.00	1.38	1.67	2.75	2.67	1.33
1.75	2.50	1.66	2.75	2.83	2.25	2.33	1.67
2.75	2.00	2.58	2.00	2.00	2.00	3.33	3.00
2.50	1.00	1.75	1.25	1.67	2.25	2.67	1.67
1.75	4.00	2.75	3.00	2.17	3.25	3.00	2.33
1.75	2.50	2.33	2.00	1.33	2.75	2.00	3.00
3.25	4.00	3.25	2.88	2.00	2.25	1.67	3.00
1.75	2.50	2.08	2.88	2.67	3.50	3.00	1.67
1.75	3.50	2.42	3.63	2.50	3.00	3.00	2.33
2.50	3.00	2.58	2.25	2.17	3.25	3.33	3.33
2.25	2.50	2.25	1.88	3.00	3.00	1.67	3.00
2.75	3.00	2.33	3.88	2.33	1.00	3.33	1.33
1.75	2.50	2.08	2.75	3.00	3.00	3.00	3.00
1.00	1.00	1.40	1.38	1.00	1.25	2.00	1.00
2.25	2.00	1.92	1.88	2.67	2.25	2.33	2.67
3.25	2.50	2.75	3.25	3.67	3.25	3.00	3.33
3.00	4.00	2.67	2.50	2.00	3.50	4.00	3.00
1.25	1.00	1.33	1.00	1.50	3.50	2.00	3.00
2.50	3.00	2.20	1.88	2.67	2.75	2.67	2.67
1.50	2.50	1.67	2.63	1.50	2.50	2.33	1.00
3.75	4.00	3.67	3.63	3.33	3.25	4.00	1.00
2.00	2.00	2.08	1.25	1.83	1.75	3.67	4.00
2.50	2.50	2.42	2.63	2.50	3.25	3.00	4.00
3.00	1.50	2.25	2.63	2.67	2.25	2.00	2.67
2.00	2.50	2.08	2.25	1.33	3.50	2.67	4.00
3.00	4.00	3.58	3.88	1.67	4.00	3.00	3.67
1.50	2.00	2.50	2.00	2.33	1.25	3.33	2.00
3.25	2.50	2.70	2.13	2.00	2.75	2.67	2.67
3.00	4.00	2.50	1.63	2.17	1.50	1.00	2.33
3.00	3.00	2.33	1.13	3.00	3.00	2.33	3.00
1.00	2.50	1.33	2.38	1.50	2.00	2.33	1.00
3.50	4.00	3.25	3.50	2.00	3.50	3.00	1.67
2.00	3.00	2.08	2.13	2.50	2.25	3.00	4.00
3.25	3.00	2.58	3.13	2.17	3.00	3.67	1.67
1.00	3.00	2.25	2.13	4.00	2.50	3.00	4.00

1.75	2.50	1.50	3.38	1.83	2.25	3.00	2.00
1.50	1.00	1.92	1.63	3.50	1.50	2.67	3.00
2.25	3.50	2.42	3.63	3.00	3.00	2.00	1.00
1.50	2.00	2.08	2.50	3.17	2.75	3.33	3.00
2.75	2.50	2.42	2.38	2.83	2.75	2.67	2.33
1.75	2.50	1.75	2.88	3.50	1.75	2.00	1.00
3.50	3.00	3.00	3.63	2.17	3.25	3.67	1.67
1.75	1.00	1.83	2.50	1.50	3.00	3.00	1.67
3.50	2.50	2.83	1.88	3.50	3.50	2.67	1.33
3.25	4.00	3.25	1.88	1.83	1.25	2.67	1.00
1.00	3.50	2.42	2.63	2.83	1.50	2.67	2.00
1.75	1.50	1.92	2.13	2.17	2.50	2.00	2.00
1.75	2.00	2.00	2.63	3.00	1.75	2.67	1.00
1.00	2.50	1.75	1.00	1.33	1.75	2.00	1.00
2.25	3.00	2.00	2.50	3.33	1.75	2.33	2.33
1.25	2.00	1.25	1.38	1.50	1.25	2.00	1.33
2.50	1.00	1.50	2.38	2.00	2.00	3.00	4.00
1.00	4.00	2.50	4.00	1.50	1.00	2.00	1.00
3.25	4.00	3.42	3.75	1.33	2.75	3.00	2.33
1.25	1.00	1.17	1.75	2.50	2.25	2.67	3.00
1.00	1.00	1.50	3.38	3.00	1.75	3.00	1.33
2.00	2.00	2.25	2.13	3.00	3.25	3.00	2.33
1.75	3.50	2.67	2.50	2.83	1.50	2.67	2.00
1.25	4.00	2.17	2.50	1.50	1.00	2.00	1.00
3.00	4.00	2.50	2.50	1.83	2.75	2.67	1.33
3.50	4.00	3.33	2.88	2.17	3.25	3.33	2.67
1.00	2.50	2.00	1.38	2.50	2.50	2.67	3.33
3.25	4.00	3.25	3.63	1.67	2.50	4.00	1.67
1.75	2.50	1.83	1.63	3.17	3.50	3.00	4.00
2.00	1.00	2.08	2.00	1.67	1.25	3.33	3.00
3.25	4.00	2.50	2.13	2.50	1.75	3.00	4.00
2.50	2.50	2.75	1.38	4.00	1.00	2.00	1.00
1.00	2.50	1.83	2.00	1.33	2.50	2.67	1.00
1.50	4.00	1.92	4.00	3.50	4.00	3.00	1.00
1.75	2.50	2.25	2.88	2.00	1.00	3.00	1.00
3.00	3.50	2.50	3.50	2.33	3.50	4.00	1.67
2.00	2.50	1.83	2.63	2.00	3.50	3.00	1.00
1.00	3.00	2.25	2.13	4.00	2.50	3.00	4.00
2.00	3.00	2.83	2.38	1.67	4.00	3.00	3.67
3.50	4.00	3.42	2.88	2.17	3.25	3.33	2.67
2.25	3.00	2.58	3.00	3.17	2.75	3.00	2.33
1.25	1.00	1.42	1.50	3.50	2.00	2.33	4.00
2.50	2.50	2.42	2.13	2.67	1.75	2.00	2.00
2.00	2.00	1.83	2.13	2.33	3.00	2.67	4.00
1.50	3.00	1.75	2.63	1.83	2.75	2.33	2.00
2.00	2.50	2.00	2.00	2.33	2.00	2.33	2.00
1.75	2.50	1.50	3.00	1.83	2.50	3.00	2.00

2.00	3.00	2.00	2.00	2.50	3.50	2.00	2.67
2.25	2.50	2.25	2.63	2.17	2.75	3.00	2.67
2.75	3.00	2.67	2.38	1.67	2.75	2.00	3.33
1.75	3.50	2.67	2.75	2.67	2.25	2.67	1.00
1.75	2.50	1.75	2.63	1.33	2.00	2.33	1.00
2.25	2.50	2.42	3.25	3.00	4.00	3.00	4.00
2.50	3.50	2.92	3.13	2.67	2.50	3.00	1.00
2.50	3.50	2.50	3.38	3.17	2.50	4.00	1.33
4.00	1.00	2.33	2.63	2.50	4.00	4.00	1.00
2.50	4.00	2.75	3.63	3.50	4.00	4.00	3.67
2.75	2.50	2.42	3.50	3.17	4.00	3.67	2.67
1.00	4.00	2.25	4.00	1.50	1.00	3.00	1.00
1.75	2.00	2.00	1.75	1.33	1.50	1.67	1.00
2.50	3.00	2.75	2.25	2.00	2.50	2.67	1.00
2.75	1.00	1.83	3.25	1.50	2.00	2.67	2.00
2.25	2.50	2.42	3.25	3.00	4.00	3.00	4.00
2.25	2.00	2.33	1.88	3.00	2.50	2.33	2.00
2.75	3.00	2.83	2.88	2.33	2.00	2.67	1.00
2.25	3.00	2.42	2.88	3.17	3.25	4.00	3.33
2.25	1.00	2.00	2.13	3.00	3.50	1.67	2.00
2.00	2.00	1.75	2.38	2.33	3.25	2.33	1.67
3.25	2.50	3.50	3.13	1.50	4.00	4.00	1.00
2.25	3.00	2.42	2.75	3.00	3.25	4.00	3.33
1.75	2.00	1.50	1.00	1.50	1.25	1.67	1.33
2.50	3.50	3.00	3.38	3.67	4.00	3.33	3.00
