# Supplementary material for: Parental-perceived home and neighborhood environmental correlates of accelerometer-measured physical activity among school-going children in Uganda
Source: PLOS Glob Public Health. 2021 Dec 8;1(12):e0000089. doi: 10.1371/journal.pgph.0000089 (PMC10021676; doi:10.1371/journal.pgph.0000089)
Supplement: Attachment — Submitted filename: Rebutal Letter.docx [file pgph.0000089.s004.docx]

**Rebuttal Letter**

We are grateful to the editor and the three reviewers for their feedback on our manuscript titled: Parental perceived home and neighbourhood environmental correlates of accelerometer-measured physical activity among school-going children in Uganda.

Please find below our response to each point raised by the academic editor and reviewers. We have edited the manuscript, defined, and consistently used abbreviations and addressed all typing errors. We have also included some new references (2017-2021) and deleted old ones with similar results, described the study area, sample selection procedure, and improved on our recommendations.

We have addressed all the concerns and our manuscript is ready for publication.

Many Thanks,

On behalf of all the authors,

Bernadette Nakabazzi

# *Academic Editor*

***Please review your reference list to ensure that it is complete and correct. If you have cited papers that have been retracted, please include the rationale for doing so in the manuscript text, or remove these references and replace them with relevant current references. Any changes to the reference list should be mentioned in the rebuttal letter that accompanies your revised manuscript. If you need to cite a retracted article, indicate the article’s retracted status in the References list and also include a citation and full reference for the retraction notice.***

**The references below have been added.**

1) Ding D, Lawson KD, Kolbe-Alexander TL, Finkelstein EA, Katzmarzyk PT, van Mechelen W, Pratt M; Lancet Physical Activity Series 2 Executive Committee. The economic burden of physical inactivity: a global analysis of major non-communicable diseases. Lancet. 2016 Sep 24;388(10051):1311-24. doi: 10.1016/S0140-6736(16)30383-X. Epub 2016 Jul 28. PMID: 27475266.
2) World Health Organization. Global action plan on physical activity 2018– 2030: more active people for a healthier world. Geneva: World Health Organization; 2018.
3) Day K. Physical Environment Correlates of Physical Activity in Developing Countries: A Review. J Phys Act Health. 2018 1;15(4):303-314. doi: 10.1123/jpah.2017-0184.

**The references below have been removed (they are old and contain same results as those in the manuscript)**

1. Committee on Environmental Health. The built environment: Designing communities to promote physical activity in children. Pediatrics 2009; 123:1591–1598. [https://doi.org/10.1542/peds.2009-0750 PMID:19482771](https://doi.org/10.1542/peds.2009-0750%20PMID:19482771).
2. Oliveira AF, Moreira C, Abreu S, Mota J, Santos R. Environmental determinants of physical activity in children: a systematic review. Arch Exercise Health Dis. 2014; 4(2):254-61. http://doi.org/10.5628/aehd. v4i2.158
3. Ding D, Sallis JF, Kerr J, Lee S, Rosenberg DE. Neighbourhood environment and physical activity among youth a review. Am J Prev Med. 2011;41(4):442-455. doi: 10.1016/j.amepre.2011.06.036
4. Davison KK, Lawson CT. Do attributes in the physical environment influence children's physical activity? A review of the literature. Int J Behav Nutr Phys Act. 2006; 3:19. doi:10.1186/1479-5868-3-19

# Reviewer # 1

***This paper presents a cross sectional study on built environment correlates of objectively measured physical activity among children in Uganda. The paper is very well written and would be useful to public health professionals and policy makers. The findings will help inform the development of effective physical activity interventions and policies. Congratulations! I am offering some suggestions for consideration before publication.***

***Abstract***

1. ***Given the scope of the journal and the physical inactivity issue, I would recommend starting the abstract with a sentence highlighting the significance of physical inactivity problem and how it is related to non-communicable diseases.***

We thank the reviewer for bringing this to our attention. We have revised the revised the starting sentence in the abstract to read as follows.

“The benefits of regular participation in Physical activity (PA) for children’s health and wellbeing are well established. However, an alarming proportion of children do not meet the PA recommendations, increasing their risk for overweight and obesity; and in turn increasing the incidence of non-communicable diseases (NCDs). Built environment characteristics in homes and neighbourhoods may constraint children’s ability to engage in PA”

***Introduction
Excellent introduction! Some minor comments below for improvement.***

2. ***Line 50: please define the acronym PA.***

We thank the reviewer for pointing this out. We have defined the acronym PA (line 56 in the marked copy of the revised manuscript)

3. ***Line 53: please define the acronym MVPA***
4. ***Please define the MVPA (i.e., using METs)***

For feedback 3 and 4; We have defined the acronym as moderate to vigorous physical activity (MVPA) (Line 59-60) in the marked up revised manuscript.

5. ***I would recommend including some more supporting statistics about the issue and the burden associated with it (e.g., healthcare, or societal costs of physical inactivity).***

We thank the reviewer for calling our attention to this, we have included the health care costs of physical inactivity line 60 to 64 in the marked-up copy of the manuscript.

6. ***The authors used objective measure for PA (i.e., accelerometer), which is excellent. I would recommend that you highlight the current lack of objectively measured PA research in LMIC compared to self-reported (which is subject to reporting bias) and that this study addresses this gap.***

We are gratefull to the reviewer for this important suggestion, we have included a statement to this effect (refer to line 123-125 in the marked up revised version of the manuscript)

***Materials and methods***

7. ***I suggest that the authors include a section for study setting/location to provide a brief description of the place/area/neighbourhood where this study was conducted.***

We thank the reviewer for this important suggestion, and we have included a brief description of the study area in the revised manuscript as to read,

“Kampala comprises five administrative divisions (Central, Nakawa, Rubaga, Kawempe and Makindye) covering an area of 189 km^2^ and a population of 1.5 million inhabitants (Uganda Bureau of Statistics (UBOS), 2018)”.

8. ***May I ask about the approach you adopted to recruit the sample?***

We used a multistage sampling method; we have explained the recruitment procedure in the marked-up copy of the revised manuscript (refer to line 139 – 147)

***Discussions***

9. ***A unique finding in this study is that the influence of the home & neighbourhood environments on Ugandan children’s PA may be gender specific. I suggest reflecting more on this by, for example, discussing the concept of embodiment which is relevant here (i.e., how Ugandan girls embody the social environment where they live and the associated gendered norms that restrict their PA participation). This is a major issue that extends to Western countries and can explain the inequities and low PA participation rates among immigrant girls (from LMICs) compared to boys in Western countries.***

Thank you for pointing this out we have included more information explaining the gender role in the relationship in line 351-360 in the revised marked up copy.

***Conclusion***

10. ***Line 351: Please correct to “environments on Ugandan children’s PA”.***

Thanks to the reviewer for suggesting this correction, we have made the necessary changes (line 389 in the marked up revised version)

11. ***I would suggest that the authors add additional recommendation highlighting*** ***the need for qualitative studies to understand the perceptions and barriers to PA engagement among girls in LMICs. This can provide more insight into the required needs to best design equitable interventions.***

We thank the reviewer for this suggestion and have added a recommendation in line 410 to 412 reading “Future qualitative studies exploring the perceptions, facilitators and barriers to PA participation among girls in LMICs are important since girls exhibit lower levels of PA than boys”. In the marked up revised version.

# Reviewer #2

***The study analyzes the Uganda context and aims to explore the relationship between home and neighborhood environment (as perceived by parents) and moderate to vigorous physical activity. The manuscript is relevant, well-written, and scientifically sound. The topic fits within the scope of the Journal.***

1. ***I consider the introduction is clear and covers a general background. An apparent gap has been identified in this section. However, I believe it would be more relevant if the authors could include more recent work (papers published in 2017-2021) since it could increase the interest of potential readers.***

We thank the reviewer for pointing out this, we have included some new references and deleted some old ones that had similar findings to be specific older review studies. (References 4, 5 and 27]

2. ***Since the research is highly focused on Kampala (Uganda) population, I consider the authors should include a description of the characteristics of the city and its environment. I would help readers out of Uganda to better understand the research results. The sample is a limitation of the study and the authors have stated it in the limitations section. For that reason, the sample characteristics explanation gains importance.***

We thank the reviewer for pointing out this important area, the section on design and participant recruitment has been revised to include a description of the study area and a description of how the sample was generated (see marked up copy of revised manuscript lines 126 to 155).

***In the methods section, the authors clearly state the eligibility criteria. Were there any exclusion criteria? Please, include them, if applicable.***

We have included the exclusion criteria for this study as children in boarding schools (line 150 in the marked up revised manuscript)

3. ***In my view, the results and the discussion are clear and understandable. However, in Table 3, in file “play equipment at home”, the “overall” result shows p>0.001. I wonder if this is a typo error and should be p<0.001 instead. Please confirm this and correct it, if applicable.***

Many thanks, this was a typing error, we have corrected this to p<0.001

4. ***The conclusions could emphasize the practical implications of the study. This way, the article would be potentially meaningful for professionals in the field, even policymakers.***

Thank you for this feedback. We have included the practical implications as requested in line 410 -414 in the revised marked up copy of the manuscript.

***The effort made by the authors is appreciated and the comments are expected to be useful.***

Thanks for appreciating our efforts and for the feed back it greatly improved our manuscript.

# Reviewer #3:

***Please, find some comments and suggestions for the manuscript entitled: “Parental perceived home and neighborhood environmental correlates of accelerometer-measured physical activity among school-going children in Uganda”. I would like to congratulate Authors for this interesting study. Nevertheless, I consider there are some small changes that should be done.***

1. ***Line 98: The abbreviation of the PA has been used in the manuscript earlier. Please, use only the abbreviation.***
2. ***Line 102: The abbreviation of the LICs has been used in the manuscript earlier. Please, use only the abbreviation.***

For feedback 1 and 2 above, we thank the reviewer for pointing out this and we have consistently used abbreviations in the revised manuscript (Lines 120 and 298 for PA and line 125 For LIC)

3. ***Line 192: Why do you think that the type of school may differentiate the results (private vs. public school)? How were the schools selected based on SES? Could you describe that process?***

We thank the reviewer for inquiring on the selection process, we have clarified on this in (refer to marked up revised version line 141-145)

4. ***Line 201 (in general all tables): I suggest removing the horizontal lines in tables (or even only some of them), however, it should stick to the Journal style (it refers to all tables in the manuscript).***

We thank the reviewer for helping us improve our tables, we have removed some of the horizontal lines as requested in all tables.

5. ***Line 256: The abbreviation of the PA has been used in the manuscript earlier. Please, use only the abbreviation.***
6. ***Line 353: The same as above.***

For feedback 5 and 6 above, we thank the reviewer for pointing out this and we have consistently used the abbreviation PA in the revised manuscript
